# Selective acceleration of disfavored enolate addition reactions by anion–π interactions†
†Electronic supplementary information (ESI) available: Detailed procedures and results for all reported experiments. See DOI: 10.1039/c5sc02563j


**DOI:** 10.1039/c5sc02563j

**Published:** 2015-08-25

**Authors:** Yingjie Zhao, Sebastian Benz, Naomi Sakai, Stefan Matile

**Affiliations:** a Department of Organic Chemistry , University of Geneva , Geneva , Switzerland . Email: stefan.matile@unige.ch ; http://www.unige.ch/sciences/chiorg/matile/ ; Fax: +41 22 379 5123 ; Tel: +41 22 379 6523

## Abstract

In chemistry and biology, cation–π interactions contribute significantly to many important transformations. In sharp contrast, reactions accomplished with support from the complementary anion–π interactions are essentially unknown. In this report, we show that anion–π interactions can determine the selectivity of the enolate chemistry of malonate half thioesters. Their addition to enolate acceptors is central in natural product biosynthesis but fails without enzymes because non-productive decarboxylation dominates. The newly designed and synthesized anion–π tweezers invert this selectivity by accelerating the disfavored and decelerating the favored process. The discrimination of anionic tautomers of different planarization and charge delocalization on π-acidic surfaces is expected to account for this intriguing “tortoise-and-hare catalysis.” Almost exponentially increasing selectivity with increasing π acidity of the catalyst supports that contributions from anion–π interactions are decisive.

## Abstract 2

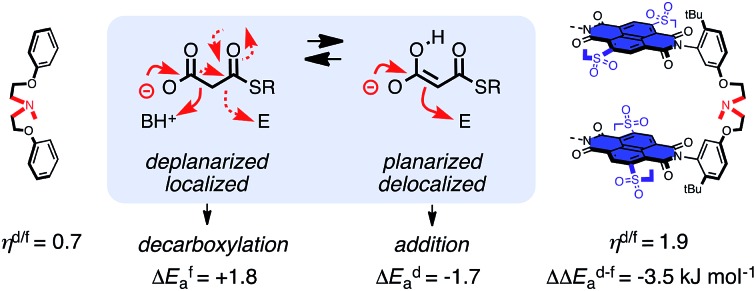
The tortoise and the hare: anion–π interactions are reported to selectively accelerate the intrinsically disfavored addition of malonate half thioesters.

## 

Cation–π interactions play a central role in molecular recognition, translocation and transformation.^1^–^5^ Arguably the most spectacular manifestation of cation–π catalysis in biology is found in the biosynthesis of steroids, in which cascade cyclization occurs *via* carbocation hopping on a stabilizing cluster of π-basic amino acid residues (Fig. 1).^2^ Cation–π interactions are also increasingly recognized in organocatalysis.^3^–^5^ Contributions from the complementary but much younger^6^ anion–π interactions^7^ have been reported for anion binding^8^–^11^ and transport.^11^,^12^ In sharp contrast, explicit considerations of anion–π interactions in catalysis are extremely rare and very recent.^13^–^15^

**Fig. 1:**
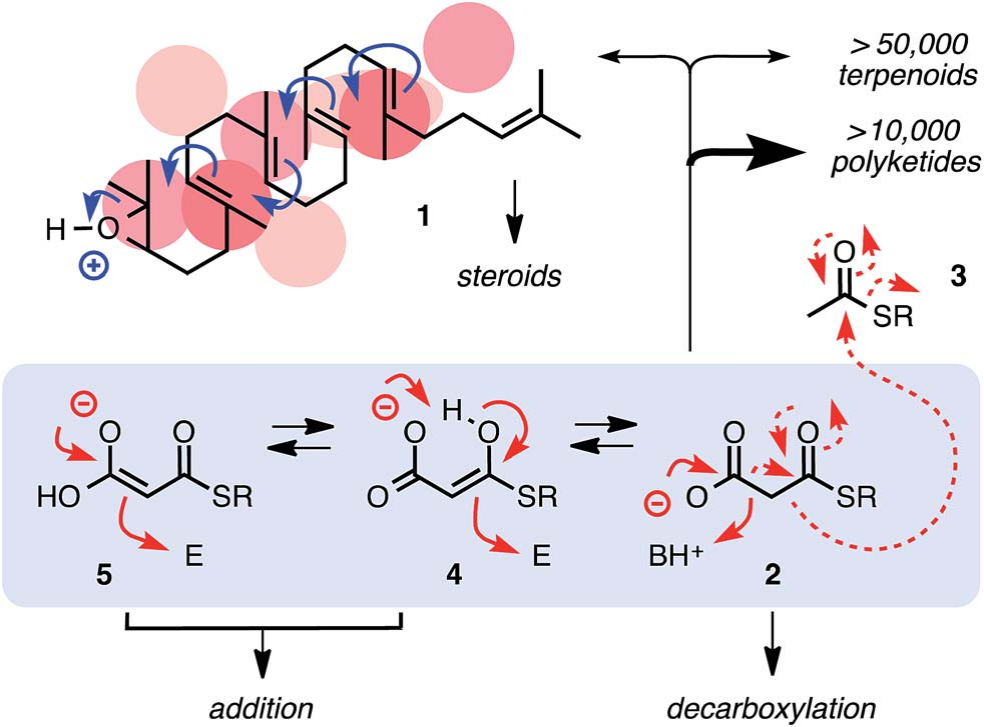
In nature, carbocation chemistry in the biosynthesis of terpenes and steroids is accomplished with cation–π interactions (red circles indicate the position of π-basic amino-acid residues in the cation–π enzyme for substrate **1**). The complementary enolate chemistry in polyketide biosynthesis and the beginning of both pathways fails in solution because decarboxylation of **2** (solid arrows) dominates over enolate addition (dashed arrows). In this report, selective acceleration of this disfavored but relevant process is achieved with anion–π interactions (blue background) and explained with the discrimination between non-planar tautomers (**2**) and planar tautomers (**4**/**5**; BH^+^: protonated base, E = electrophilic carbon).

Looking for more significant transformations that could benefit from anion–π interactions, we considered malonate half thioester (MHT) **2**, which is obtained by deprotonation of malonyl-CoA, a malonic acid half thioester (MAHT), under mildest conditions (Fig. 1).^16^ Claisen condensation with acetyl-CoA **3** yields acetoacetyl-CoA as entry into both biosynthetic pathways, terpenoids and polyketides. Catalyzed by polyketide synthases, repeated decarboxylative enolate addition of the same substrate provides access to more than 10 000 natural products as important as fatty acids and lipids, macrolactones and higher aromatics.^16^ In solution, MHTs have been shown to add to nitroolefins, enones, aldehydes, imines or (thio)esters as in polyketide biosynthesis.^17^–^22^

However, under unoptimized conditions, the non-productive decarboxylation to **3** dominates. Recent mechanistic studies indicate that for addition to occur, it should precede decarboxylation.^21^,^22^ This should be possible with tautomers **4** or **5**, whereas tautomer **2** should favor decarboxylation. Control over the selectivity between addition and decarboxylation thus calls for the discrimination between planar tautomers in which the negative charge is delocalized by resonance and tautomers in which planarity and resonance are disrupted by the tetrahedral sp^3^ carbon between the two carbonyl groups. Anion recognition on π-acidic aromatic planes appeared just ideal to feel these subtle structural differences.

The addition of aromatic^18^ and aliphatic MAHTs **6** and **7** to aromatic and aliphatic nitroolefins^9^–^12^,^20^ **8** and **9** was selected to elaborate on this hypothesis (Fig. 2). In bifunctional anion–π catalysts, π-acidic 1,4,5,8-naphthalenediimide (NDI) derivatives^12^–^14^,^23^–^25^ are appended to an amine base to provide stabilizing π surfaces for the enolate intermediates as soon as they are produced. All substrates and catalysts were synthesized from commercially available starting materials in a few steps (Scheme S1†).^26^ The reaction of MAHTs **6** and **7** with nitroolefins **8** and **9** was followed by ^1^H NMR spectroscopy, in which the evolution of the intrinsically disfavored addition products d (**10**) and the favored decarboxylation products f (**3**) was recorded with time against internal standards (Fig. S1–S4†). Results were quantified with *η*^d/f^ values, that is the yield *η*^d^ of the intrinsically disfavored divided by *η*^f^ for the favored product (Table 1).

**Fig. 2:**
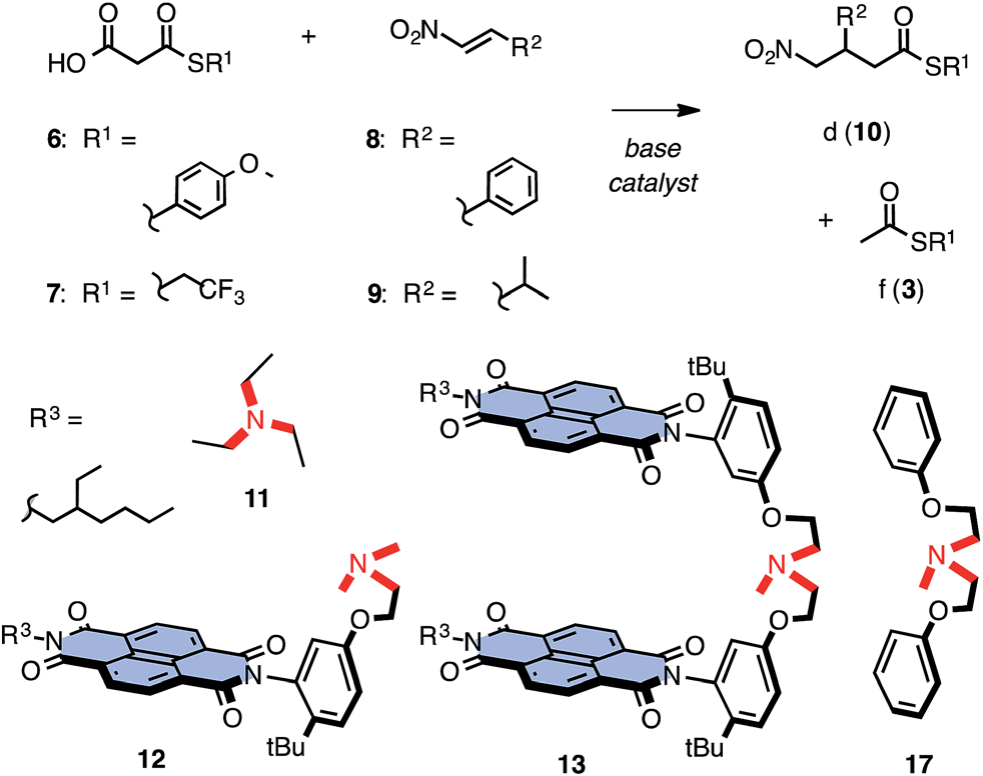
Structure of substrates (**6–9**), favored product f (**3**), disfavored product d (**10**), the minimalist bifunctional catalyst **12**, anion–π tweezer **13** and control bases **11** and **17**.

**Table 1:** Characteristics of anion–π catalysts and controls^*a*^

	C^*b*^	*E*_LUMO_ ^*c*^ (eV)	S^1^^*b*^	S^2^^*b*^	S^1^/C^*d*^	*T* (°C)^*e*^	*t* (h)^*f*^	*η* ^d^ ^*g*^ (%)	*η* ^f^ ^*h*^ (%)	*η* ^d/f^ ^*i*^	Δ*E*da^*j*^ (kJ mol^–1^)	Δ*E*fa^*k*^ (kJ mol^–1^)	ΔΔ*E*d–fa^*l*^ (kJ mol^–1^)
1	**11**	—	**6**	**8**	0.2	RT	1.5	36	62	0.6	—	—	—
2	**12**	–4.2	**6**	**8**	0.2	RT	6	46	54	0.8	+2.8/–3.5^*m*^	+3.8/–2.7^*m*^	–0.9/–0.8^*m*^
3	**13**	–4.2	**6**	**8**	0.2	RT	15	48	51	0.9	+5.5/–0.8^*m*^	+6.6/+0.1^*m*^	–1.1/–0.9^*m*^
4	**14**	–3.9	**6**	**8**	0.2	RT	12	50	48	1.0	+4.5/–1.8^*m*^	+5.6/–0.9^*m*^	–1.1/–0.9^*m*^
5	**15**	–4.4	**6**	**8**	0.2	RT	12	59	36	1.6	+3.9/–2.4^*m*^	+6.1/–0.4^*m*^	–2.2/–2.0^*m*^
6	**16**	–4.6	**6**	**8**	0.2	RT	12	59	31	1.9	+3.7/–2.7^*m*^	+6.6/+0.1^*m*^	–2.9/–2.6^*m*^
7	**17**	—	**6**	**8**	0.2	RT	24	37	53	0.7	—	—	—
8	**11**	—	**6**	**8**	0.2	5	8	57	40	1.4	—	—	—
9	**12**	–4.2	**6**	**8**	0.2	5	20	69	30	2.3	+1.3/–3.3^*m*^	+2.5/–2.6^*m*^	–1.2/–0.7^*m*^
10	**13**	–4.2	**6**	**8**	0.2	5	40	77	20	3.8	+3.7/–0.9^*m*^	+5.8/+0.7^*m*^	–2.1/–1.6^*m*^
11	**14**	–3.9	**6**	**8**	0.2	5	40	71	23	3.1	+3.7/–0.9^*m*^	+5.4/+0.3^*m*^	–1.8/–1.2^*m*^
12	**15**	–4.4	**6**	**8**	0.2	5	40	80	14	5.7	+3.1/–1.4^*m*^	+6.6/+1.5^*m*^	–3.5/–2.9^*m*^
13	**16**	–4.6	**6**	**8**	0.2	5	40	80	11	7.3	+2.8/–1.7^*m*^	+6.9/+1.8^*m*^	–4.1/–3.5^*m*^
14	**16**	–4.6	**6**	**8**	0.02	5	672	84	14	6.0	—	—	—
15	**17**	—	**6**	**8**	0.2	5	75	60	30	2.0	—	—	—
16	**11**	—	**6**	**9**	0.2	5	9	40	54	0.7	—	—	—
17	**13**	–4.2	**6**	**9**	0.2	5	70	59	40	1.5	+3.1	+4.3	–1.3
18	**13**	–4.2	**6**	**9**	0.2	RT	20	31	65	0.5	+5.1/–1.0^*m*^	+8.1/+0.9^*m*^	–3.0/–1.9^*m*^
19	**14**	–3.9	**6**	**9**	0.2	RT	20	28	62	0.4	+5.3/–0.8^*m*^	+7.6/+0.5^*m*^	–2.3/–1.3^*m*^
20	**15**	–4.4	**6**	**9**	0.2	RT	20	39	53	0.7	+4.7/–1.4^*m*^	+8.4/+1.2^*m*^	–3.7/–2.6^*m*^
21	**16**	–4.6	**6**	**9**	0.2	RT	20	45	48	0.9	+4.4/–1.8^*m*^	+8.5/+1.4^*m*^	–4.2/–3.2^*m*^
22	**17**	—	**6**	**9**	0.2	RT	25	23	70	0.3	—	—	—
23	**13**	–4.2	**7**	**8**	0.2	RT	9	41	57	0.7	–0.7	+0.5^*m*^	–1.2^*m*^
24	**17**	—	**7**	**8**	0.2	RT	9	33	66	0.5	—	—	—

^*a*^Reactions were conducted in THF, with 4–40 mM catalyst C, 200 mM substrate S^1^ (**6** and **7**), 2 M S^2^ (**8** and **9**), results were analyzed by ^1^H NMR spectroscopy, compare Fig. 3a and 4 for data analysis.

^*b*^See Fig. 2 and 3 for structures.

^*c*^LUMO energy levels in eV relative to –5.1 eV for Fc^+^/Fc, approximated from cyclic voltammetry data.^13^,^23^,^25^

^*d*^Catalyst C per substrate S^1^ used in the reaction.

^*e*^Reaction temperature, RT = room temperature.

^*f*^Reaction time for >95% conversion.

^*g*^Yield of intrinsically disfavored product d (**10**).

^*h*^Yield of intrinsically favored product f (**3**).

^*i*^ *η* ^d/f^ = *η*^d^/*η*^f^.

^*j*^Difference in activation energy of the disfavored (d) reaction compared to control **11** (or **17**)^*m*^, from initial velocity of formation of product d (**10**).

^*k*^Same for favored (f) reaction *vs.***11** (or **17**)^*m*^, from *v*_ini_ of f (**3**).

^*l*^Selective catalysis: Δ*E*da – Δ*E*fa.

^*m*^Measured against **17**.

Catalyzed with TEA **11** at room temperature, *η*^d/f^ = 0.6 confirmed that the undesired decarboxylation is indeed favored under these conditions (Fig. 2, Table 1, entry 1). In comparison, the simplest possible anion–π catalyst, *i.e.*, catalyst **12** composed of a π-acidic NDI surface next to a tertiary amine base, already gave rise to a slightly better *η*^d/f^ = 0.8 (Table 1, entry 2). The number of π-acidic surfaces in the catalyst was doubled next to increase the effective molarity of π-acidic surfaces or to even act from two sides on the reaction. The resulting *η*^d/f^ = 0.9 demonstrated that with anion–π tweezer **13**, addition became almost as good as decarboxylation (Table 1, entry 3).

These encouraging results called for a systematic assessment of the contribution from anion–π interactions. The reversible oxidation of sulfide donors to sulfoxide and sulfone acceptors has been introduced and validated previously as unique approach to vary π acidity with minimal structural changes.^18^ Anion–π tweezer **14** with two sulfides in the core of each NDI was prepared as a mixture of axial stereoisomers (Fig. 3b). The temperature-controlled stepwise sulfide oxidation was unproblematic as long as the tertiary amine was protected first against oxidation by protonation with TFA. Although insufficient,^7^ the energy levels of the LUMOs are used as an approximative measure for π acidity. They decrease from *E*_LUMO_ = –3.9 eV for NDIs in **14** with two sulfide donors to *E*_LUMO_ = –4.4 eV for NDIs in **15** with sulfoxide acceptors and *E*_LUMO_ = –4.6 eV for NDIs in **16** with sulfones.^12^,^13^ With decreasing *E*_LUMO_ of the catalyst, the selectivity increased almost exponentially14*b* from *η*^d/f^ = 1.0 for **14** to *η*^d/f^ = 1.6 for **15** and *η*^d/f^ = 1.9 for the strongest π acid **16** (Fig. 3a, [black circle]; Table 1, entries 4–6). Enolate addition became more dominant at lower temperatures. At 5 °C, selectivity perfectly followed π acidity, increasing from *η*^d/f^ = 3.1 for anion–π tweezers **14** with donating sulfides to *η*^d/f^ = 3.8 for tweezers **13** with unsubstituted NDIs and *η*^d/f^ = 5.7 and *η*^d/f^ = 7.3 for tweezers **15** and **16** with withdrawing sulfoxides and sulfones, respectively (Fig. 3a, ♦; Table 1, entries 10–13).

**Fig. 3:**
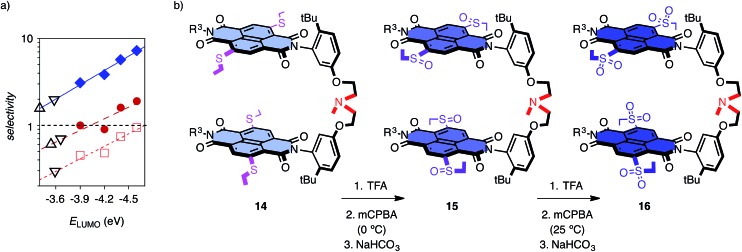
(a) Dependence of *η*^d/f^, *i.e.* the yield *η*^d^ of the intrinsically disfavored product (**10**) divided by *η*^f^ of the favored product (**3**), on the energy of the LUMO of tweezers **13–16** at RT (red, [black circle], □) and 5 °C (blue, ♦) for substrates **6** ([black circle], ♦) and **7** (□) with **8**; with exponential curve fit. Controls **11** (△) and **17** (▽) select below *E*_LUMO_ = –3.7 eV of π-neutral NDIs. (b) Stepwise oxidation of the core substituents of anion–π tweezers **14** gradually increases the π acidity of the catalyst without global structural changes. All tweezers used are mixtures of stereoisomers (axial chirality, sulfoxides).^25^

All reactions proceeded to completion, with little formation of other side products (Table 1). The nitroolefin acceptor **8** was used in excess to maximize the probability of addition once the reactive enolate is formed on the π-acidic surface. For comparative evaluation, 20 mol% catalyst was used with regard to the MAHT substrate **6**. With the best anion–π tweezer **16**, selectivity ratios were with *η*^d/f^ = 6.0 nearly preserved at reduced catalyst loading (Table 1, entries 13 and 14). With 2 mol% **16**, full conversion within 30 days at 5 °C gave a turnover number TON = 50 (Fig. S2†).

Replacement of the π-basic phenyl substituents in substrates **6** and **8** by alkyl groups in **7** and **9** did not disturb the observed trends (Fig. 2, Table 1, entries 16–24). With **6** and **9** at low temperature, a clean inversion of selectivity was obtained from control **11** with preference for decarboxylation (*η*^d/f^ = 0.7) to anion–π tweezers **13** with preference for addition (*η*^d/f^ = 1.5, Table 1, entries 16 and 17). Measured at RT, *η*^d/f^ values increased with increasing π acidity of the catalyst from *η*^d/f^ = 0.4 for **14** with sulfide donors to *η*^d/f^ = 0.5 for original **13** and *η*^d/f^ = 0.7 and *η*^d/f^ = 0.9 for **15** and **16** with increasing π acidity (Fig. 3a, □; Table 1, entries 18–21). As a final control, we replaced TEA **11** by a standard more similar to the operational anion–π tweezers **12–16**. With substrates **6** and **8** at room temperature, control **17** afforded *η*^d/f^ = 0.7 (Fig. 3a, ▽; Table 1, entry 7), better than TEA **11** (*η*^d/f^ = 0.6, Fig. 3a, △) but clearly inferior to the original tweezers **13** (*η*^d/f^ = 0.9, Table 1, entry 3) and far off the best performing anion–π tweezers **16** (*η*^d/f^ = 1.9, Table 1, entry 6; Fig. 3a, [black circle]).

The dependence of selectivity on π acidity, expressed as *E*_LUMO_ of the catalysts, was close to exponential,14*b* independent of temperature and substrates (Fig. 3a, [black circle], ♦, □). The compared to the perfect sulfur series **14–16** somewhat underperforming unsubstituted NDI tweezers **13** indicated the presence of minor, supportive as well as constant contributions from the ethyl sidechains to catalysis and thus confirmed the importance of the isostructural variation of π acidity provided by stepwise sulfide oxidation in the series **14–16** (Fig. 3a and 4). The selectivities obtained for controls **11** and **17** at different temperatures clustered below a virtual *E*_LUMO_ = –3.7 eV (Fig. 3a, △, ▽). This value corresponds to a nearly π-neutral NDI with two alkylamino donors in the core.^12^,^23^ Selectivity values of controls coinciding with those extrapolated for π-neutral NDI surfaces provided corroborative support that anion–π interactions indeed account for the selective acceleration of disfavored reactions.

**Fig. 4:**
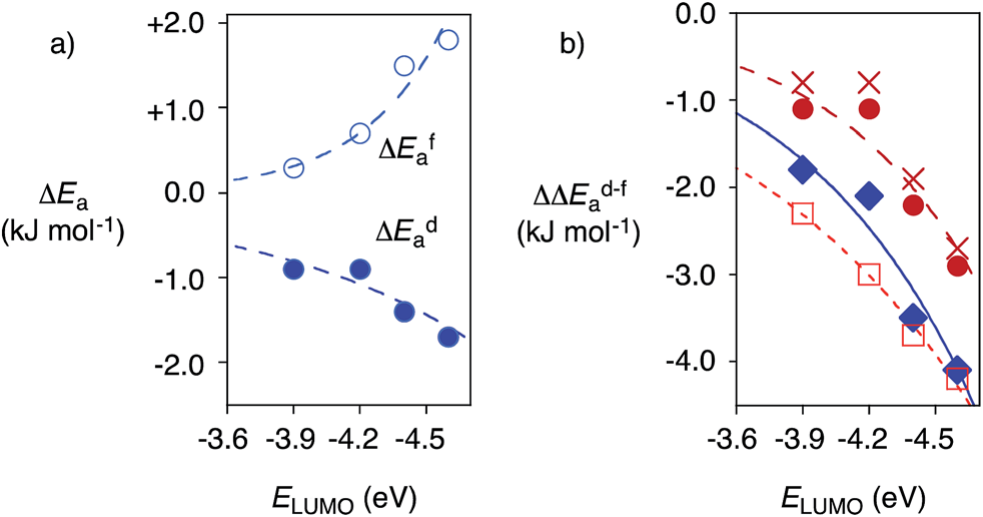
(a) Dependence of the changes in activation energy Δ*E*_a_ for substrate **6** for the favored decarboxylation (Δ*E*fa, ○) and the disfavored addition (to **8**, Δ*E*da, [black circle]) on the π acidity of anion–π tweezers **13–16** (*E*_LUMO_), relative to control **17**, at 5 °C, with exponential curve fit (Table 1, entries 10–13). (b) Selective acceleration of a disfavoured reaction: dependence of ΔΔ*E*d–fa, *i.e.*, Δ*E*da – Δ*E*fa, for **6** on the π acidity of **13–16** (*E*_LUMO_) compared to control **11** ([black circle], ♦, □) or **17** (X) at RT ([black circle], X, □) and 5 °C (♦) for **6** ([black circle], X, ♦) and **7** (□) with **8**, with exponential curve fit (compare Table 1).

Reactions with anion–π catalysts were slower than with TEA **11** but still much faster than without any amine catalyst. The initial velocities of product formation were used to determine activation energies *E*fa and *E*da, that is the energy difference between ground state and transition state for the favored decarboxylation (f) and the disfavored addition (d, Fig. S5†). Subtraction of activation energies of controls **11** or **17** from those of anion–π catalysts gave Δ*E*fa and Δ*E*da (deceleration: Δ*E*_a_ > 0, acceleration: Δ*E*_a_ < 0). Positive Δ*E*fa and Δ*E*da revealed that compared to control **11**, anion–π tweezers **13–16** slowed down both processes (Table 1). Compared to the more revealing control **17**, anion–π catalysts **13–16** always accelerated the disfavored (Δ*E*da < 0) and mostly decelerated the favored process (Δ*E*fa > 0, Table 1). Most impressive the trends at low temperatures: without exception, acceleration of disfavored and deceleration of favored reaction both increased with increasing π acidity of anion–π tweezers **13–16** (Fig. 4a, Table 1, entries 10–13).

Selective acceleration of a disfavored reaction is given as ΔΔ*E*d–fa = Δ*E*da – Δ*E*fa < 0, valid for both deceleration or acceleration of the competing processes. Close to exponential14*b* increase of the negative ΔΔ*E*d–fa with increasing π acidity of the catalyst was found, independent of conditions (Fig. 4b, [black circle] (warm) *vs.* ○ (cold)), substrates (Fig. 4b, [black circle] (**6**) *vs.* □ (**7**)) and controls (Fig. 4b, [black circle] (**11**) *vs.* X (**17**), Table 1). This consistent kinetic response to increasing π acidity supported that the inversion of selectivity indeed originates from anion–π interactions.

Selective deceleration of the favored decarboxylation and selective acceleration of the disfavored addition were both in agreement with the envisioned discrimination of differently planarized and delocalized tautomers by anion–π interactions (Fig. 1). It might be important to recall that direct experimental evidence for the ground-state stabilization of enolates on π-acidic surfaces is available from covalent model systems.^14^ Transition-state destabilization for decarboxylation (by immobilizing the localized negative charge in tautomer **2** on the carboxylate oxygens on the π-acidic surface) could contribute as well. The same is true for transition-state stabilization for addition (by stabilizing the formation of the topologically matching nitronate^9^–^12^ on the π-acidic surface). More explicit comments on mechanisms, applications and perspectives^26^,13c would be premature. Such concluding remarks are also not needed to appreciate the main lesson learned from this study: selective “tortoise-and-hare catalysis”^27^ of enolate chemistry provides experimental support that anion–π catalysis^13^ not only exists but also matters.

## Supplementary Material

Supplementary information
