# Supplementary material for: Selective acceleration of disfavored enolate addition reactions by anion–π interactions
Source: Chem Sci. 2015 Aug 25;6(11):6219–23. doi: 10.1039/c5sc02563j (PMC6054047; doi:10.1039/c5sc02563j)
Supplement: Supplementary file 1 [file SC-006-C5SC02563J-s001.pdf]

Electronic Supplementary Material (ESI) for Chemical Science.
This journal is © The Royal Society of Chemistry 2015

Selective acceleration of disfavored enolate addition reactions by anion-π interactions

Yingjie Zhao, Sebastian Benz, Naomi Sakai and Stefan Matile*

Department of Organic Chemistry, University of Geneva, Geneva, Switzerland

**Corresponding author. E-mail: stefan.matile@unige.ch*

Supporting information

1. Supplementary methods

1.1. Materials and methods

As in reference 13. Reagents for synthesis were purchased from Sigma-Aldrich, Fluka, Acros, Apollo Scientific and Bachem. All reactions were performed under N_2 or Ar atmosphere. Unless stated otherwise, column chromatography was carried out on silica gel 60 (Fluka, 40-63 μm). Analytical (TLC) and preparative thin layer chromatography (PTLC) were performed on silica gel 60 (Fluka, 0.2 mm) and silica gel GF (Analtech, 1 mm), respectively. Melting points (Mp) were measured on a Melting Point M-565 (BUCHI). UV-Vis spectra were recorded on a JASCO V-650 spectrophotometer equipped with a stirrer and a temperature controller (25 °C) and are reported as maximal absorption wavelength λ in nm (extinction coefficient ε in $M^{-1}cm^{-1}$). IR spectra were recorded on a Perkin Elmer Spectrum One FT-IR spectrometer (ATR, Golden Gate, unless stated) and are reported as wavenumbers ν in cm^{-1} with band intensities indicated as s (strong), m (medium), w (weak). ^1H and ^{13}C spectra were

recorded (as indicated) either on a Bruker 300 MHz, 400 MHz or 500 MHz spectrometer and are reported as chemical shifts (δ) in ppm relative to TMS (δ = 0). Spin multiplicities are reported as a singlet (s), doublet (d), triplet (t) and quartet (q) with coupling constants (*J*) given in Hz, or multiplet (m). Broad peaks are marked as br. ^{1}H and ^{13}C resonances were assigned with the aid of additional information from 1D & 2D NMR spectra (H,H-COSY, DEPT 135, HSQC and HMBC). ESI-MS were performed on a Finnigan MAT SSQ 7000 instrument or a ESI API 150EX and are reported as *m/z* (%). Accurate mass determinations using ESI (HR ESI-MS) were performed on a Sciex QSTAR Pulsar mass spectrometer.

Abbreviations. AcOH: Acetic acid; EtOAc: Ethyl acetate; DCM: Dichloromethane; DMF: *N*,*N*-Dimethylformamide; mCPBA: 3-Chloroperbenzoic acid; MeOH: Methanol; rt: Room temperature; TFA: Trifluoroacetic acid; THF: Tetrahydrofuran.

1.2. Synthesis

Compound 6. This compound was prepared following the literature procedure.[18]

Compound 9. This compound was prepared following the literature procedure.[S1]

Compound 18. This compound was prepared following the literature procedure.[S2]

Br,Br-NDA. This compound was prepared following the literature procedure.[S3]

Compound 20. This compound was prepared following the literature procedure.[S4]

Compounds 21, 22. These compounds were prepared following the literature procedure.[S5]

Compound 7. Malonyl chloride (500 mg, 0.34 mL, 3.5 mmol) and 2,2,2-Trifluorethanthiol (410 mg, 0.32 mL, 3.5 mmol) were stirred in 5 mL dry THF under an argon atmosphere at 0 ℃ for 6 hours. The reaction was quenched by the addition of saturated aqueous $NaHCO_3$, and extracted with ether. The organic layer was discarded, the aqueous layer was acidified to pH 1 with 2 M HCl and extracted with EtOAc, dried over Na_2SO_4. The yellow solution was

concentrated *in vacuo* and purified by column chromatography (1:1 Pentane/EtOAc + 2% AcOH) to yield **7** (212 mg, 35%) as a viscousoil. TLC (1:1 Pentane/EtOAc + 2% AcOH): R_f 0.54; IR (neat): 2942 (w), 1692 (s), 1397 (w), 1237 (m), 1085 (s), 999 (m), 915 (m), 845 (m), 639 (s); ^{1}H NMR (400 MHz, $CDCl_3$): 3.72 (s, 2H), 3.66 (q, ^{3}J (H,F) = 9.7 Hz, 2H); ^{13}C NMR (101 MHz, $CDCl_3$): 187.4 (*C*), 170.0 (*C*), 124.5 (*C*) (q, ^{1}J (C,F) = 275.9 Hz), 48.3 (*CH_2*), 31.2 (*CH_2*) (q, ^{2}J (C,F) = 34.7 Hz); MS (ESI, +ve): 171 (100, $[M+H]^+$).

Compound 19. A solution of **18** (379 mg, 1.0 mmol) and **20** (198 mg, 1.2 mmol) in DMF (5 mL) was heated to reflux for 30 hours. The resulting mixture was poured into 1 M HCl (50 mL), and the precipitate was collected by filtration and washed with water. Silica gel column chromatography of the residue (DCM/acetone 30:1 then 20:1) gave **19** (268 mg, 51%) as a yellow solid. TLC (DCM/acetone 20:1): R_f 0.29; Mp: 161 – 162 ºC; IR (neat): 2958 (w), 1706 (m), 1661 (s), 1580 (m), 1452 (m), 1334 (s), 1245 (s), 1192 (m), 769 (s); ^{1}H NMR (400 MHz, $CDCl_3$): 8.75 – 8.67 (m, 4H), 7.38 (d, ^{3}J (H,H) = 8.9 Hz, 1H), 6.77 (dd, ^{3}J (H,H) = 8.9 Hz, ^{4}J (H,H) = 2.7 Hz, 1H), 6.37 (d, ^{4}J (H,H) = 2.7 Hz, 1H), 4.13 – 4.01 (m, 2H), 1.91 – 1.82 (m, 1H), 1.34 – 1.20 (m, 8H), 1.14 (s, 9H), 0.90 – 0.78 (m, 6H); ^{13}C NMR (101 MHz, $CDCl_3$): 163.9 (*C*), 163.2 (*C*), 155.0 (*C*), 138.5 (*C*), 132.7 (*C*), 131.6 (*CH*), 131.1 (*CH*), 130.4 (*CH*), 127.1 (*C*), 126.9 (*C*), 126.5 (*C*), 117.3 (*CH*), 117.2 (*CH*), 44.7 (*CH_2*), 38.1 (*CH_3*), 35.3 (*C*), 31.8 (*CH_3*), 30.9 (*CH*), 30.7 (*CH_2*), 28.6 (*CH_2*), 24.0 (*CH_2*), 23.1 (*CH_2*), 14.1 (*CH_3*), 10.6 (*CH_3*); MS (ESI, +ve): 471 (100, $[M–C_4H_8+H]^+$), 527 (25, $[M+H]^+$), 1071 (10, $[2M+NH_4]^+$); HRMS (ESI, +ve) calcd for $C_{32}H_{35}N_2O_5$: 527.2541, found: 527.2559.

Compound 17. A solution of **22** (200 mg, 0.85 mmol) and Phenol (160 mg, 1.7 mmol) in CH_3CN (15 mL) was heated to 180 ℃ in a pressure tube for 12 hours. The resulting dark solution was concentrated *in vacuo* and purified by column chromatography (9:1 DCM/MeOH) to yield **17** (170 mg, 37%) as a light brown oil. TLC (DCM/MeOH 9:1): R_f 0.67; IR (neat): 2943 (w), 1707 (m), 1666 (s), 1582 (m), 1452 (m), 1334 (m), 1243 (s), 1034 (m), 768 (s); ^{1}H NMR (400 MHz, $CDCl_3$): 7.33 – 7.28 (m, 4H), 7.01 – 6.88 (m, 6H), 4.14 (t, ^{3}J (H,H) = 5.8 Hz,

4H), 2.99 (t, ^3J (H,H) = 5.8 Hz, 4H), 2.52 (s, 3H); ^{13}C NMR (101 MHz, $CDCl_3$): 158.7 (*C*), 129.4 (*CH*), 120.8 (*CH*), 114.6 (*CH*), 66.0 (*CH_2*), 56.6 (*CH_2*), 43.5 (*CH_3*); MS (ESI, +ve): 272 (100, $[M+H]^+$); HRMS (ESI, +ve) calcd for $C_{17}H_{22}NO_2$: 272.1645, found: 272.1646.

Compound 13. A solution of **19** (331 mg, 0.63 mmol) and **22** (59 mg, 0.25 mmol) in CH_3CN (20 mL) was heated to 180 ºC in a pressure tube for 24 hours. The resulting mixture was concentrated *in vacuo.* Silica gel column chromatography of the residue (DCM/acetone 10:1 then 5:1) gave **13** (130 mg, 46%) as a yellow solid. TLC (DCM/acetone 5:1): R_f 0.44; Mp: 161 – 162 ºC; IR (neat): 2945 (w), 1707 (m), 1665 (s), 1580 (m), 1451 (m), 1333 (s), 1244 (s), 1191 (m), 768 (s); ^{1}H NMR (400 MHz, $CDCl_3$): 8.72 (s, 8H), 7.47 (d, ^3J (H,H) = 9.0 Hz, 2H), 6.93 (d, ^3J (H,H) = 9.0 Hz, 2H), 6.51 (s, 2H), 4.17 – 4.02 (m, 4H), 4.05 – 3.94 (m, 4H), 2.86 (s, br, 4H), 2.36 (s, br, 3H), 1.98 – 1.78 (m, 2H), 1.40 – 1.19 (m, 16H), 1.15 (s, 18H), 0.92 – 0.78 (m, 12H); ^{13}C NMR (101 MHz, $CDCl_3$): 163.7 (*C*), 163.2 (*C*), 157.0 (*C*), 139.2 (*C*), 133.1 (*C*), 131.4 (*CH*), 131.1 (*CH*), 130.2 (*CH*), 127.1 (*C*), 127.0 (*C*), 126.7 (*C*), 116.2 (*CH*), 116.0 (*CH*), 65.9 (*CH_2*), 56.2 (*CH_2*), 44.7 (*CH_2*), 43.1 (*CH*), 38.0 (*CH_3*), 35.4 (*C*), 31.8 (*CH_3*), 30.7 (*CH_2*), 28.6 (*CH_2*), 24.1 (*CH_2*), 23.1 (*CH_2*), 14.1 (*CH_3*), 10.6 (*CH_3*); MS (ESI, +ve): 1137 (100, $[M+H]^+$), 2273 (10, $[2M+H]^+$); HRMS (ESI, +ve) calcd for $C_{69}H_{78}N_5O_{10}$: 1136.5743, found: 1136.5776.

Compound 12. A solution of **19** (263 mg, 0.50 mmol) and **21** (147 mg, 1.0 mmol) in CH_3CN (20 mL) was heated to 180 ºC in a pressure tube for 24 hours. The resulting mixture was concentrated *in vacuo.* Silica gel column chromatography of the residue (acetone/MeOH 10:1) gave **12** (63 mg, 21%) as a yellow solid. TLC (acetone/MeOH 10:1): R_f 0.34; Mp: 111 – 112 ºC; IR (neat): 2943 (w), 1706 (m), 1664 (s), 1451 (m), 1334 (s), 1244 (s), 1191 (m), 1033 (w), 769 (s); ^{1}H NMR (400 MHz, $CDCl_3$): 8.73 (s, 2H), 8.73 (s, 2H), 7.49 (d, ^3J (H,H) = 9.0 Hz, 1H), 6.97 (dd, ^3J (H,H) = 9.0 Hz, ^4J (H,H) = 2.8 Hz, 1H), 6.53 (d, ^4J (H,H) = 2.8 Hz, 1H), 4.15 – 4.03 (m, 2H), 4.00 (t, ^3J (H,H) = 5.7 Hz, 2H), 2.67 (t, ^3J (H,H) = 5.7 Hz, 2H), 2.26 (s, 6H), 1.82 – 1.93 (m, 1H), 1.40 – 1.21 (m, 8H), 1.17 (s, 9H), 0.88 (t, ^3J (H,H) = 7.4 Hz, 3H),

0.82 (t, ^{3}J (H,H) = 6.9 Hz, 3H); ^{13}C NMR (101 MHz, $CDCl_3$): 163.7 (*C*), 163.2 (*C*), 157.5 (*C*), 139.1 (*C*), 133.1 (*C*), 131.4 (*CH*), 131.1 (*CH*), 130.1 (*CH*), 127.1 (*C*), 127.0 (*C*), 126.7 (*C*), 116.2 (*CH*), 116.0 (*CH*), 65.8 (CH_2), 58.1 (CH_2), 45.7 (CH_3), 44.6 (CH_2), 38.0 (*CH*), 35.4 (*C*), 31.8 (CH_3), 30.7 (CH_2), 28.6 (CH_2), 24.0 (CH_2), 23.1 (CH_2), 14.1 (CH_3), 10.6 (CH_3); MS (ESI, +ve): 598 (100, $[M+H]^+$); HRMS (ESI, +ve) calcd for $C_{36}H_{44}N_3O_5$: 598.3276, found: 598.3289.

Compound 23. A suspension of Br,Br-NDA (4.23 g, 10 mmol), **20** (1.65 g, 10 mmol) and 2-ethylhexylamine (3.87 g, 30 mmol) in AcOH (100 mL) was heated at 80 ºC for 20 hours. The resulting mixture was diluted with DCM, washed with brine, dried over $MgSO_4$ and concentrated *in vacuo*. Silica gel column chromatography of the residue (DCM to DCM/acetone 40:1 to 20:1) gave **23** (1.77 g, 26%) as an orange solid. TLC (DCM/acetone 20:1): R_f 0.44; Mp: 302 – 303 ºC; IR (neat): 2958 (w), 2861 (w), 1708 (m), 1658 (s), 1418 (m), 1299 (m), 1228 (s), 868 (m), 787 (m); ^{1}H NMR (400 MHz, $CDCl_3/CD_3OD$ 2:1): 8.92 (s, 2H), 7.41 (d, ^{3}J (H,H) = 9.0 Hz, 1H), 6.85 (d, ^{3}J (H,H) = 9.0 Hz, 1H), 6.45 (s, 1H), 4.10 – 4.07 (m, 2H), 1.90 – 1.83 (m, 1H), 1.38 – 1.19 (m, 8H), 1.13 (s, 9H), 0.87 (t, ^{3}J (H,H) = 7.5 Hz, 3H), 0.84 – 0.78 (m, 3H); ^{13}C NMR (101 MHz, $CDCl_3/CD_3OD$ 2:1): 161.9 (*C*), 161.3 (*C*), 161.1 (*C*), 155.9 (*C*), 139.1 (*CH*), 138.9 (*CH*), 137.5 (*C*), 132.8 (*C*), 129.9 (*CH*), 128.5 (*C*), 128.2 (*C*), 128.2 (*C*), 128.0 (*C*), 125.7 (*C*), 125.6 (*C*), 124.5 (*C*), 124.4 (*C*), 117.1 (*CH*), 116.7 (*CH*), 44.9 (CH_2), 37.8 (CH_3), 35.0 (*C*), 31.5 (CH_3), 30.5 (CH_2), 29.5 (*CH*), 28.4 (CH_2), 23.9 (CH_2), 22.9 (CH_2), 13.6 (CH_3), 10.2 (CH_3); MS (ESI, +ve): 629 (100, $[M–C_4H_8+H]^+$), 685 (20, $[M+H]^+$), 1388 (12, $[2M+Na]^+$); HRMS (ESI, +ve) calcd for $C_{32}H_{33}Br_2N_2O_5$: 683.0751, found: 683.0727.

Compounds 24. A solution of **23** (682 mg, 1.0 mmol), ethanethiol (10 mL, 134 mmol), K_2CO_3 (2.8 g, 20 mmol) and 18–crown–6 (80 mg, 0.3 mmol) in $CHCl_3$ (100 mL) was heated at 80 ºC for 6 hours. The mixture was purified by silica gel column chromatography directly (DCM to DCM/acetone 40:1) to give **24** (433 mg, 67%) as a red solid. TLC (DCM/acetone 20:1): R_f 0.41; Mp: 145 – 146 ºC; IR (neat): 2959 (w), 1693 (m), 1649 (s), 1546 (m), 1440 (s), 1317 (m), 1219 (s), 788 (m); ^{1}H NMR (400 MHz, $CDCl_3$): 8.63 (s, 2H), 7.43 (d, ^{3}J (H,H) = 8.9

Hz, 1H), 6.84 (dd, ^3J (H,H) = 8.9 Hz, ^4J (H,H) = 2.8 Hz, 1H), 6.44 (d, ^4J (H,H) = 2.8 Hz, 1H), 4.19 – 4.03 (m, 2H), 3.25 – 3.08 (m, 4H), 2.00 – 1.81 (m, 1H), 1.49 – 1.40 (m, 6H), 1.38 – 1.17 (m, 8H), 1.14 (s, 9H), 0.91 – 0.75 (m, 6H); ^{13}C NMR (101 MHz, $CDCl_3$): 164.0 (*C*), 163.5 (*C*), 163.4 (*C*), 162.6 (*C*), 155.1 (*C*), 149.6 (*C*), 148.7 (*C*), 138.2 (*C*), 132.7 (*C*), 130.2 (*CH*), 128.4 (*CH*), 128.3 (*CH*), 125.1 (*C*), 124.9 (*C*), 123.9 (*C*), 123.6 (*C*), 119.3 (*C*), 118.7 (*C*), 117.5 (*CH*), 117.1 (*CH*), 44.9 (CH_2), 37.8 (CH_3), 35.2 (*C*), 31.8 (CH_3), 30.9 (*CH*), 30.7 (CH_2), 28.6 (CH_2), 26.4 (CH_2), 24.0 (CH_2), 23.1 (CH_2), 14.1 (CH_3), 12.7 (CH_3), 10.6 (CH_3); MS (ESI, +ve): 591 (100, $[M–C_4H_8+H]^+$), 647 (44, $[M+H]^+$), 1311 (22, $[2M+NH_4]^+$); HRMS (ESI, +ve) calcd for $C_{36}H_{43}S_2N_2O_5$: 647.2608, found: 647.2610.

Compound 14. A solution of **24** (407 mg, 0.63 mmol) and **22** (59 mg, 0.25 mmol) in CH_3CN (20 mL) was heated to 180 ºC in a pressure tube for 24 hours. The resulting mixture was concentrated *in vacuo*. Silica gel column chromatography of the residue (DCM/acetone 20:1 then 10:1) gave **14** (165 mg, 48%) as a red solid. TLC (DCM/acetone 10:1): R_f 0.33; Mp: 180 – 181 ºC; IR (neat): 2921 (w), 1694 (m), 1651 (s), 1546 (m), 1439 (s), 1313 (m), 1222 (s), 787 (m); ^{1}H NMR (400 MHz, $CDCl_3$): 8.65 (s, 4H), 7.46 (d, ^3J (H,H) = 9.0 Hz, 2H), 6.93 (dd, ^3J (H,H) = 9.0 Hz, ^4J (H,H) = 2.8 Hz, 2H), 6.53 (d, ^4J (H,H) = 2.8 Hz, 2H), 4.17 – 4.05 (m, 4H), 3.99 (s, br, 4H), 3.23 – 3.11 (m, 8H), 2.81 (t, ^3J (H,H) = 5.7 Hz, 4H), 2.32 (s, 3H), 1.97 – 1.86 (m, 2H), 1.47 – 1.39 (m, 12H), 1.36 – 1.20 (m, 16H), 1.14 (s, 9H), 1.14 (s, 9H), 0.90 – 0.79 (m, 12H); ^{13}C NMR (101 MHz, $CDCl_3$): 164.1 (*C*), 163.8 (*C*), 163.2 (*C*), 162.8 (*C*), 157.6 (*C*), 149.4 (*C*), 148.6 (*C*), 138.9 (*C*), 133.1 (*C*), 130.0 (*CH*), 128.5 (*CH*), 125.4 (*C*), 125.2 (*C*), 124.0 (*C*), 124.0 (*C*), 119.4 (*CH*), 119.1 (*CH*), 116.3 (*C*), 116.1 (*C*), 66.2 (CH_2), 56.4 (CH_2), 44.8 (CH_2), 43.1 (*CH*), 37.8 (CH_3), 35.4 (*C*), 31.8 (CH_3), 30.7 (CH_2), 28.6 (CH_2), 26.3 (CH_2), 24.0 (CH_2), 23.1 (CH_2), 14.1 (CH_3), 12.8 (CH_3), 10.6 (CH_3); MS (ESI, +ve): 1377 (100, $[M+H]^+$), 1393 (67, $[M+NH_4]^+$); HRMS (ESI, +ve) calcd for $C_{77}H_{94}N_5O_{10}S_4$: 1376.5878, found: 1376.5833.

Compound 15. To a solution of **14** (50 mg, 0.036 mmol) in DCM (5 mL), TFA (0.5 mL)

was added at rt. The mixture was then stirred for 5 min at rt. Then DCM and TFA were removed *in vacuo* to give **25**. Without further purification, to a solution of **25** in DCM (5 mL), TFA (50 μL) was added at rt, then mCPBA (42 mg, 0.18 mmol) was added at 0 ºC. The mixture was then stirred for 1 hour at 0 ºC. The resulting mixture was washed with aqueous $Na_2S_2O_3$ (10%), brine, $NaHCO_3$ (sat.), dry over Na_2SO_4 and concentrated *in vacuo*. Silica gel column chromatography of the residue (DCM/acetone/MeOH 80:15:3) gave **15** (24 mg, 47%) as a yellow solid. TLC (DCM/acetone/MeOH 80:15:3): R_f 0.28; Mp: 310 – 311 ºC; IR (neat): 2974 (w), 1707 (m), 1658 (s), 1442 (m), 1301 (m), 1243 (s), 1051 (s), 792 (w); ^{1}H NMR (400 MHz, $CDCl_3$): 9.58 – 9.34 (m, 4H), 7.50 (d, ^3J (H,H) = 9.0 Hz, 2H), 6.97 (d, ^3J (H,H) = 9.0 Hz, 2H), 6.53 (s, 2H), 4.19 – 3.96 (m, 8H), 3.38 – 3.10 (m, 4H), 3.09 – 2.72 (m, 8H), 2.39 (s, 3H), 1.85 (s, br, 2H), 1.38 – 1.21 (m, 28H), 1.16 – 1.10 (m, 18H), 0.96 – 0.73 (m, 12H); ^{13}C NMR (101 MHz, $CDCl_3$): 162.6 (*C*), 161.9 (*C*), 161.1 (*C*), 160.6 (*C*), 156.6 (*C*), 154.9 (*C*), 154.4 (*C*), 138.1 (*C*), 131.1 (*C*), 129.4 (*CH*), 127.8 (*CH*), 127.0 (*C*), 126.8 (*C*), 125.9 (*C*), 125.5 (*C*), 122.2 (*C*), 121.9 (*C*), 115.8 (*CH*), 115.1 (*CH*), 65.1 (CH_2), 55.4 (CH_2), 47.9 (CH_2), 43.9 (CH_2), 42.1 (*CH*), 36.9 (CH_3), 34.4 (*C*), 30.8 (CH_3), 29.6 (CH_2), 27.3 (CH_2), 23.0 (CH_2), 22.1 (CH_2), 13.1 (CH_3), 9.4 (CH_3), 6.4 (CH_3); MS (ESI, +ve): 1441 (100, $[M+H]^+$); HRMS (ESI, +ve) calcd for $C_{77}H_{94}N_5O_{14}S_4$: 1440.5675, found: 1440.5611.

Compound 16. To a solution of **25** (54 mg, TFA salt, 0.036 mmol) in DCM (5 mL), TFA (50 μL) was added at rt, then mCPBA (168 mg, 0.72 mmol) was added at rt. The mixture was then stirred for 6 hours at rt. The resulting mixture was washed with aqueous $Na_2S_2O_3$ (10%), brine, $NaHCO_3$ (sat.), dry over Na_2SO_4 and concentrated *in vacuo*. Silica gel column chromatography of the residue (DCM/Acetone/MeOH 80:15:3) gave **16** (13 mg, 25%) as a yellow solid. TLC (DCM/Acetone/MeOH 80:15:3): R_f 0.44; Mp: 193 – 194 ºC; IR (neat): 2960 (w), 1675 (s), 1430 (w), 1309 (m), 1238 (m), 1133 (s), 1017(w), 712 (s); ^{1}H NMR (400 MHz, $CDCl_3$): 9.62 – 9.20 (m, 4H), 7.52 – 7.44 (m, 2H), 6.99 – 6.92 (m, 2H), 6.57 (s, 2H), 4.23 – 4.09 (m, 4H), 4.08 – 3.72 (m, 12H), 2.86 (s, br, 4H), 2.39 (s, br, 3H), 1.94 – 1.85 (m, 2H), 1.41

– 1.18 (m, 28H), 1.16 (s, 9H), 1.14 (s, 9H), 0.94 – 0.77 (m, 12H); ^{13}C NMR (101 MHz, $CDCl_3$): 160.4 (*C*), 160.3 (*C*), 159.9 (*C*), 159.9 (*C*), 156.4 (*C*), 145.0 (*C*), 144.7 (*C*), 144.6 (*C*), 138.0 (*C*), 138.0 (*C*), 133.0 (*CH*), 132.9 (*CH*), 132.7 (*CH*), 132.5 (*CH*), 131.2 (*C*), 129.2 (*CH*), 129.2 (*CH*), 128.8 (*C*), 128.7 (*C*), 128.5 (*C*), 128.5 (*C*), 126.9 (*C*), 126.8 (*C*), 126.6 (*C*), 126.5 (*C*), 126.3 (*C*), 126.2 (*C*), 125.8 (*C*), 125.8 (*C*), 115.5 (*CH*), 115.4 (*CH*), 115.4 (*CH*), 115.3 (*CH*), 64.9 (CH_2), 54.9 (CH_2), 49.7 (CH_2), 49.4 (CH_2), 49.3 (CH_2), 44.3 (CH_2), 42.2 (CH_3), 36.9 (*CH*), 34.3 (*C*), 30.8 (CH_3), 29.6 (CH_2), 27.4 (CH_2), 22.9 (CH_2), 22.1 (CH_2), 13.1 (CH_3), 9.5 (CH_3), 6.5 (CH_3); MS (ESI, +ve): 1505 (100, $[M+H]^+$); HRMS (ESI, +ve) calcd for $C_{77}H_{94}N_5O_{18}S_4$: 1504.5471, found: 1504.5468.

1.3. Anion-π catalysis and transition-state stabilization

Solutions of substrates **6** or **7** (200 mM) and **8** or **9** (2000 mM) and catalysts **11**-**17** (4 - 40 mM) were prepared in THF-d_8 and stirred at RT or 5 ºC. ^{1}H NMR of the mixture diluted in $CDCl_3$ was recorded. For the reaction between substrates **6** and **8**, **9**, the methoxyl group from the substrate and product whose proton number doesn't change was used as an internal standard (Fig. S1, S2 and S3 as representative examples). For the reaction between substrates **7** and **8**, the catalyst protons were used as an internal standard (Fig. S4 as a representative example). The concentration of the products **10** (addition) and **3** (decarboxylation) was determined from the integration of pertinent resonances. Concentrations of **10** and **3** were plotted against time, and the initial velocities were determined from the linear fitting (Fig. S5 as a representative example). From equation S1, we could get the rate constants.

$$k_{\mathrm{app}} = v_{\mathrm{ini}} / ([\mathbf{6} \text{ or } \mathbf{7}]_0\ [\mathbf{8} \text{ or } \mathbf{9}]_0) \qquad \text{S1}$$

Then the rate enhancements k_{rel} were calculated from equation S2.

$$k_{\mathrm{rel}} = k_{\mathrm{app}}\ (1) / k_{\mathrm{app}}\ (2) \qquad \text{S2}$$

Changes in activation energy ΔE_a were determined by equation S3.

$$\Delta E_a = -RT \ln k_{rel} \qquad \text{S3}$$

Finally, selective catalysis of a disfavored reaction $\Delta\Delta E_a^{d\text{-}f}$ were approximated by equation S4.

$$\Delta\Delta E_a^{d\text{-}f} = \Delta E_a^{d} - \Delta E_a^{f} \qquad \text{S4}$$

The selectivity values **10**/**3** obtained at completion of the reaction are reported in Table 1 and Fig. 3a.

1.4. References

S1 M. Weber, W. Frey and R. Peters, *Angew. Chem. Int. Ed.* 2013, **52**, 13223–13227.

S2 K. Tambara, N. Ponnuswamy, G. Hennrich and G. D. Pantoş, *J. Org. Chem.* 2011, **76**, 3338−3347.

S3 C. Thalacker, C. Röger and F. Würthner, *J. Org. Chem.* 2006, **71**, 8098−8105.

S4 U. Sheth, et al, PCT Int. Appl. 2011072241.

S5 F. Aricò, S. Evaristo and P. Tundo, *ACS Sustainable Chem. Eng*. 2013, **1**, 1319−1325.

2. Supplementary figures and legends

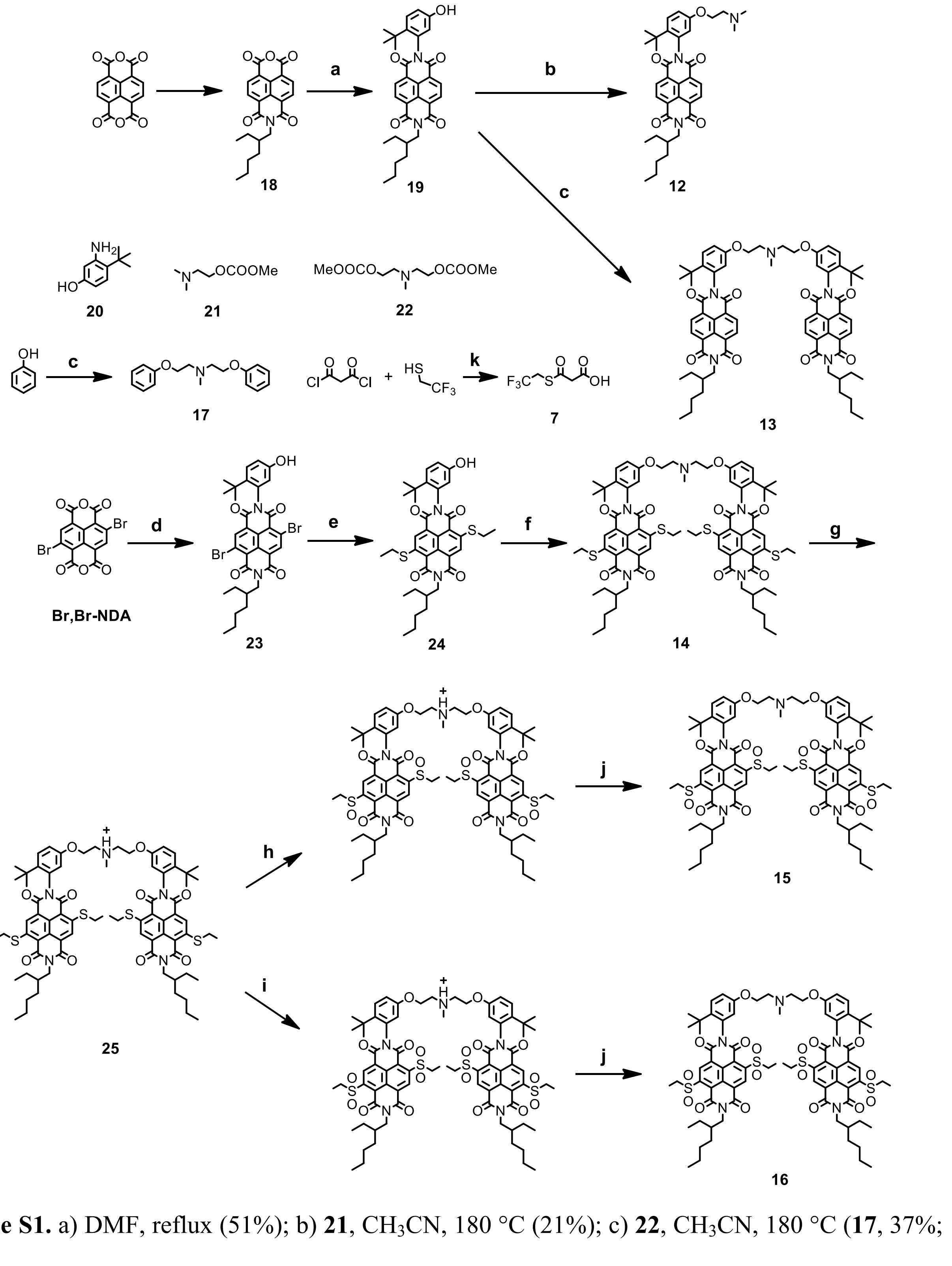

Scheme S1. a) DMF, reflux (51%); b) **21**, CH_3CN, 180 °C (21%); c) **22**, CH_3CN, 180 °C (**17**, 37%; **13**, 46%); d) **20**, 2-ethylhexylamine, AcOH, 85 °C (26%); e) ethanethiol, K_2CO_3, 18-crown-6, $CHCl_3$, 80 °C (67%); f) **22**, CH_3CN, 180 °C (48%); g) TFA, DCM; h) mCPBA, DCM, 0 °C; i) mCPBA, DCM, r.t; j) $NaHCO_3$ (3 steps, **15**, 47%; **16**, 25%); k) THF, 0 °C (35%).

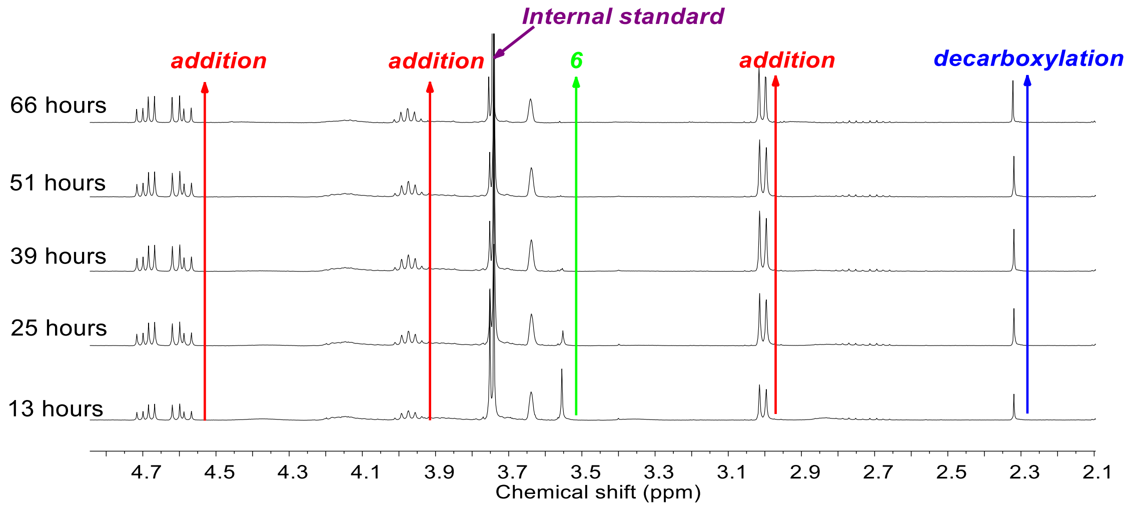

Fig. S1. ^{1}H NMR spectra of a mixture of **6** (200 mM), **8** (2 M) and catalyst **16** (40 mM) in THF–d_8 at 5 °C diluted in $CDCl_3$. The red arrows show the formation of addition product, the blue one shows the decarboxylation and the green one shows the consumption of substrate **6**. Methoxyl group is used as an internal standard (purple arrow).

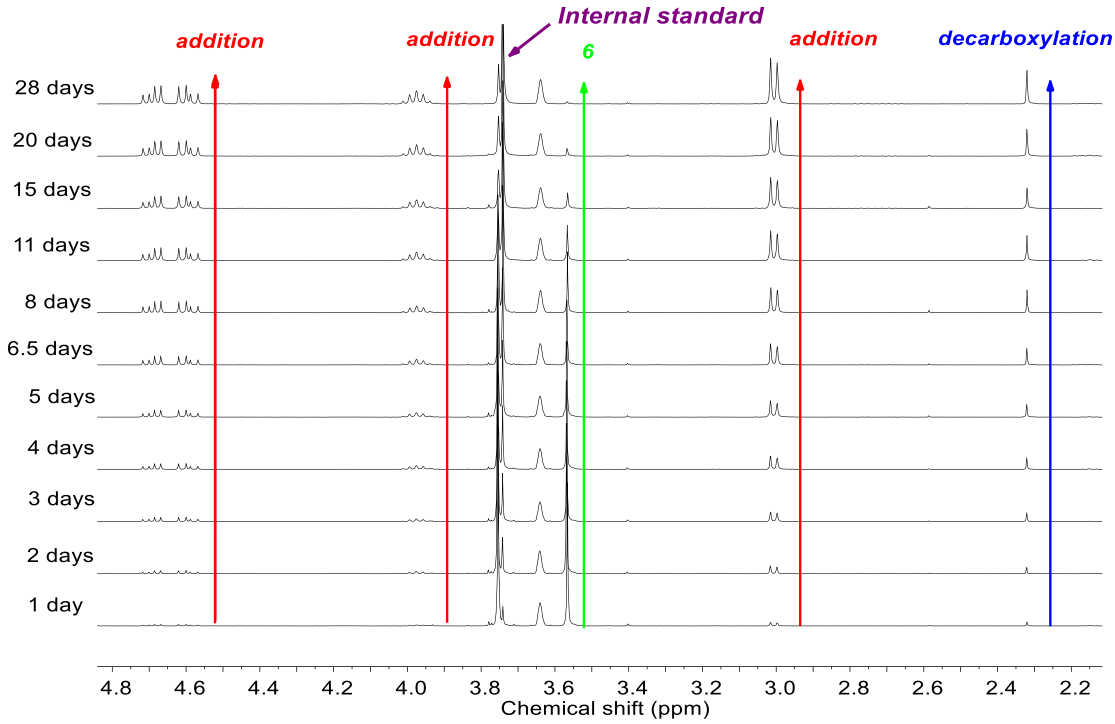

Fig. S2. ^{1}H NMR spectra of a mixture of **6** (200 mM), **8** (2 M) and catalyst **16** (4 mM) in THF–d_8 at 5 °C diluted in $CDCl_3$. The red arrows show the formation of addition product, the blue one shows the decarboxylation and the green one shows the consumption of substrate **6**. Methoxyl group is used as an internal standard (purple arrow).

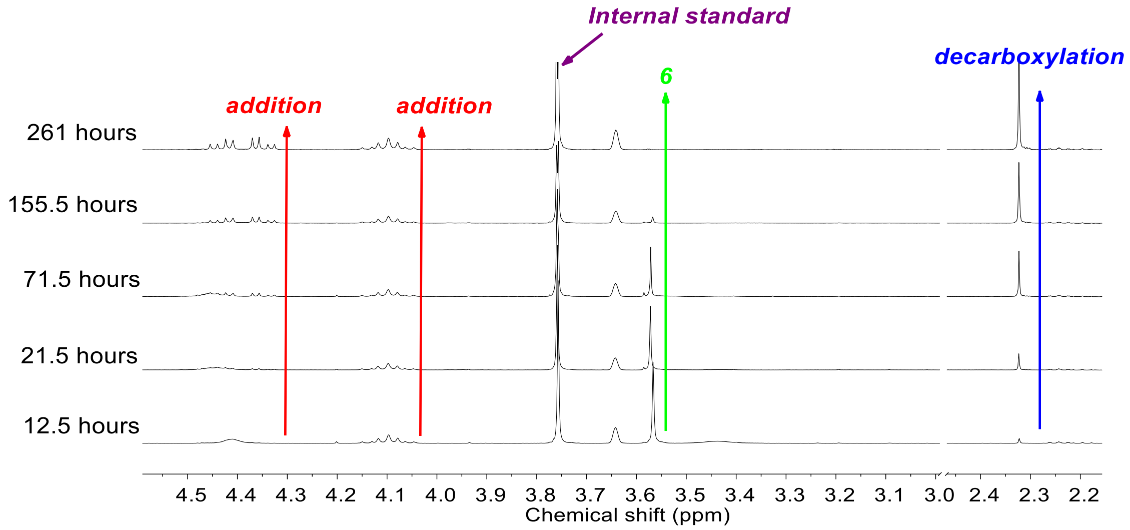

Fig. S3. ^{1}H NMR spectra of a mixture of **6** (200 mM), **9** (2 M) and catalyst **13** (40 mM) in THF–d_8 at 5 °C diluted in $CDCl_3$. The red arrows show the formation of addition product, the blue one shows the decarboxylation and the green one shows the consumption of substrate **6**. Methoxyl group is used as an internal standard (purple arrow).

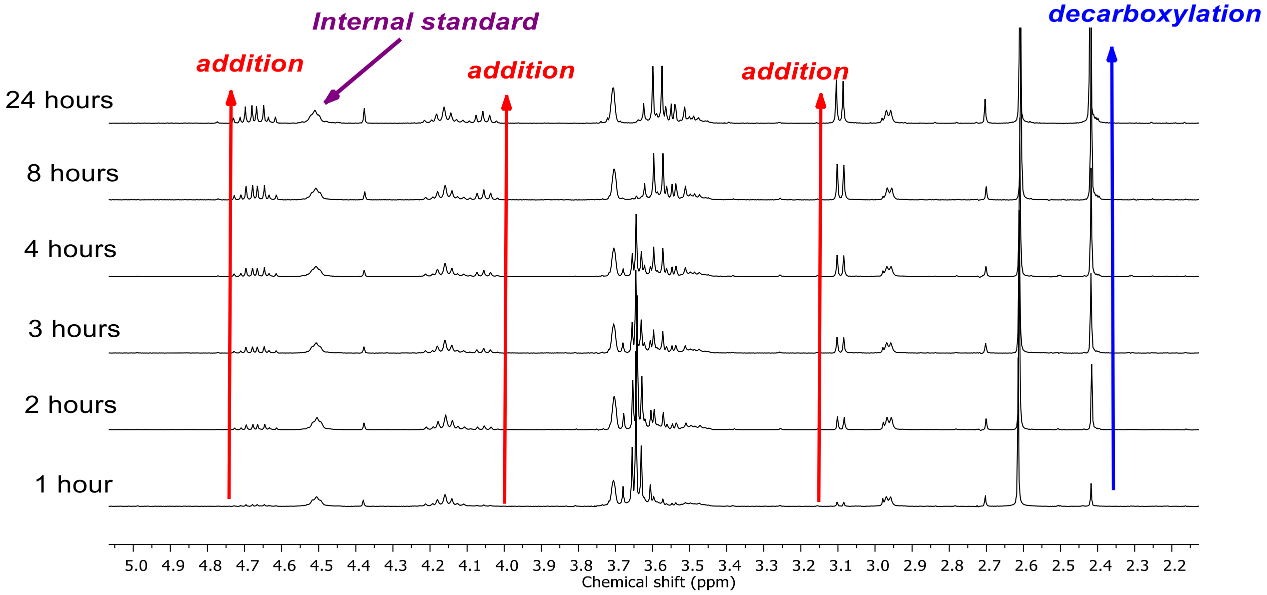

Fig. S4. ^{1}H NMR spectra of a mixture of **7** (200 mM), **8** (2 M) and catalyst **13** (40 mM) in THF–d_8 at rt diluted in $CDCl_3$. The red arrows show the formation of addition product, the blue one shows the decarboxylation. The catalyst proton is used as an internal standard (purple arrow).

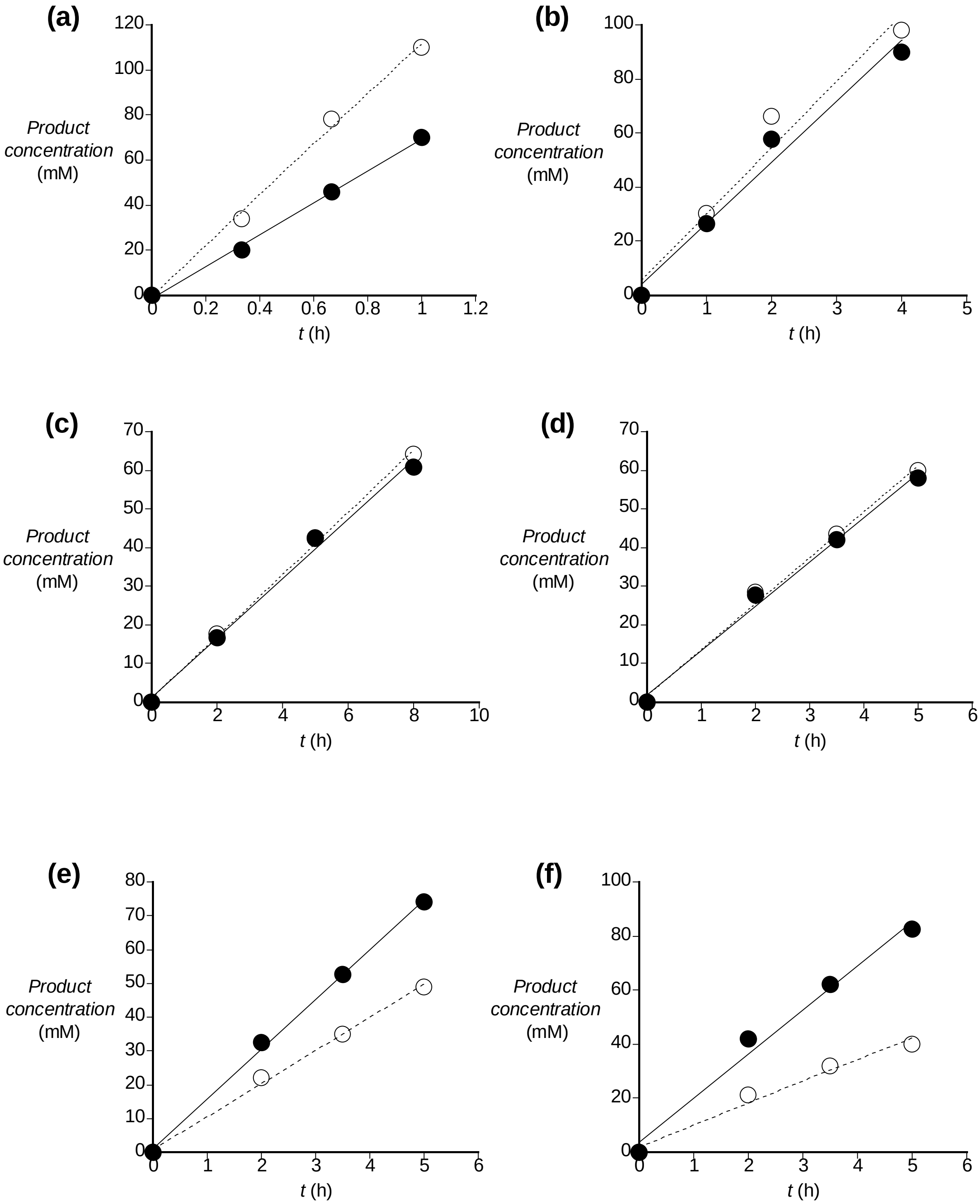

Fig. S5. Reaction kinetics for addition (filled circles) and decarboxylation (empty circles) reaction of **6** (200 mM), **8** (2 M) and catalyst **11** (a)**, 12** (b), **13** (c)**, 14** (d), **15** (e), **16** (f**)** (40 mM) in THF–d_8 at rt as representative examples. The initial velocity was determined by the linear fitting.

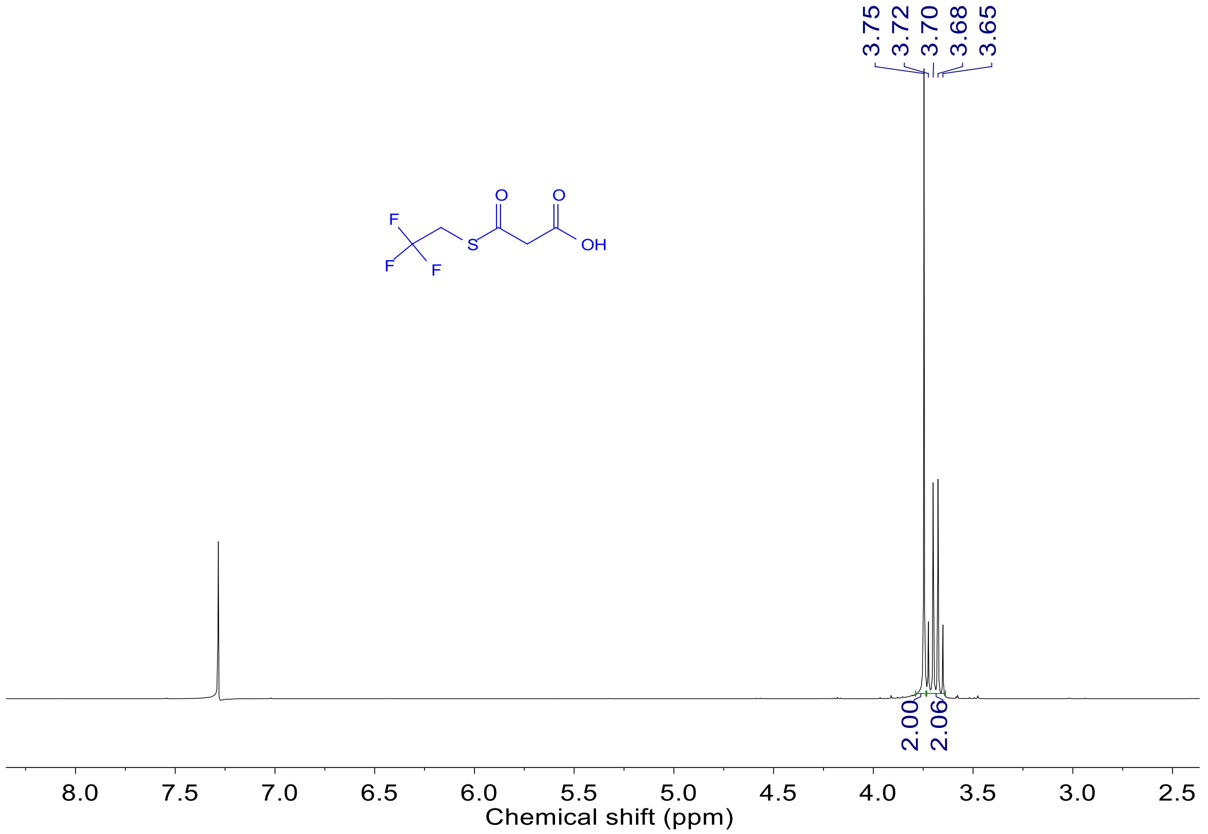

Fig. S6. ^{1}H NMR spectrum of **7** in $CDCl_3$.

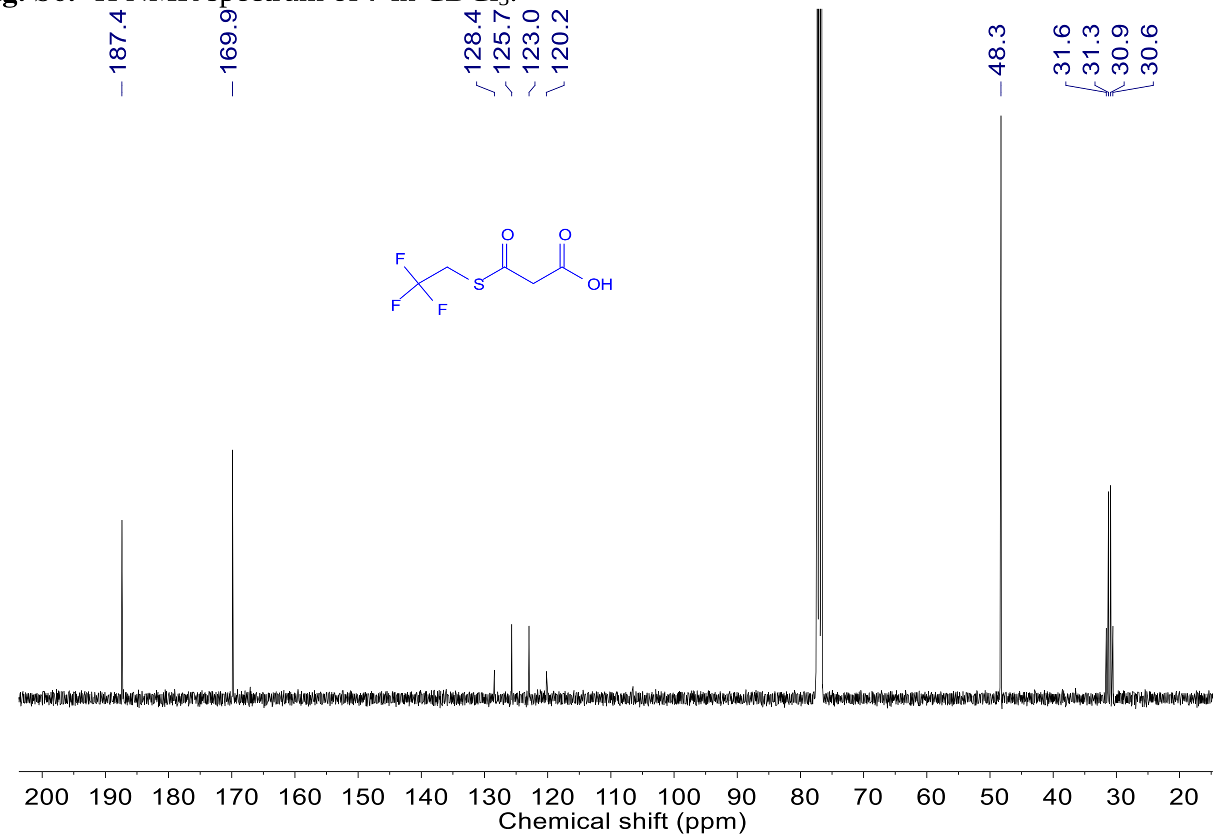

Fig. S7. ^{13}C NMR spectrum of **7** in $CDCl_3$.

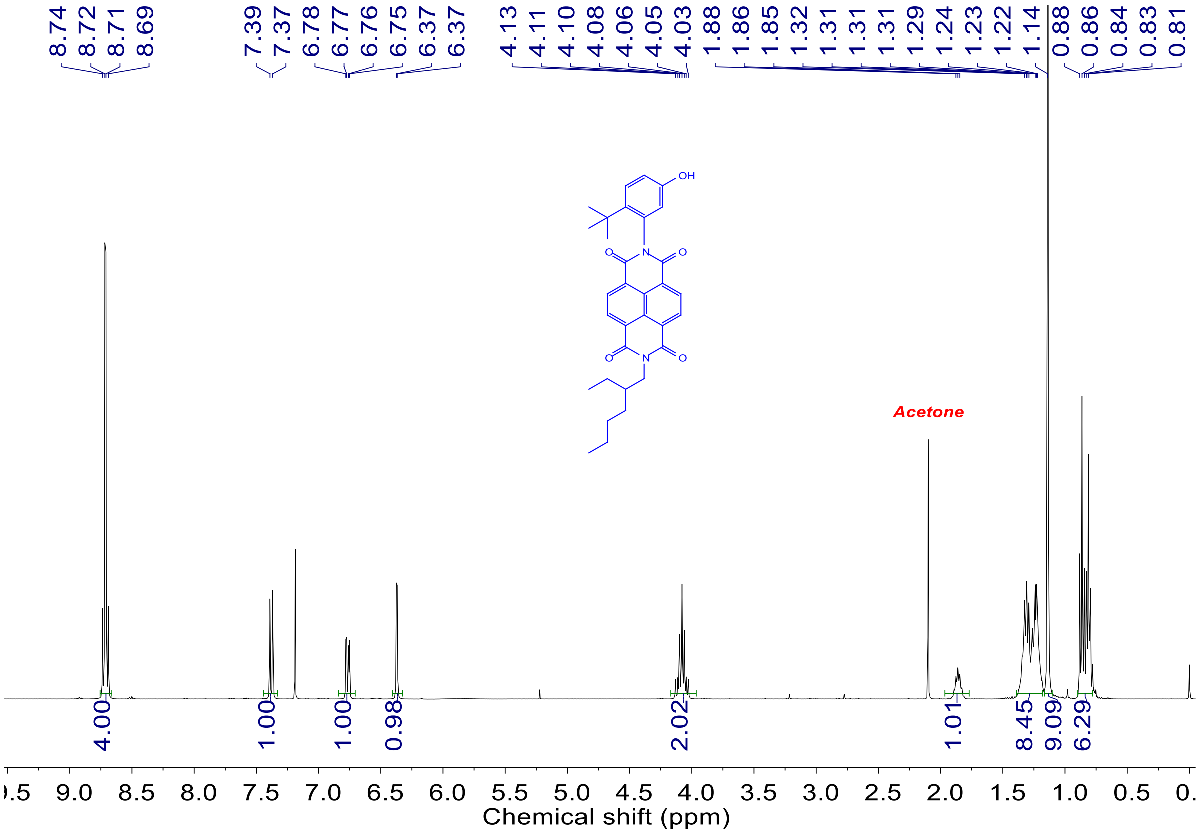

Fig. S8. ^{1}H NMR spectrum of **19** in $CDCl_3$.

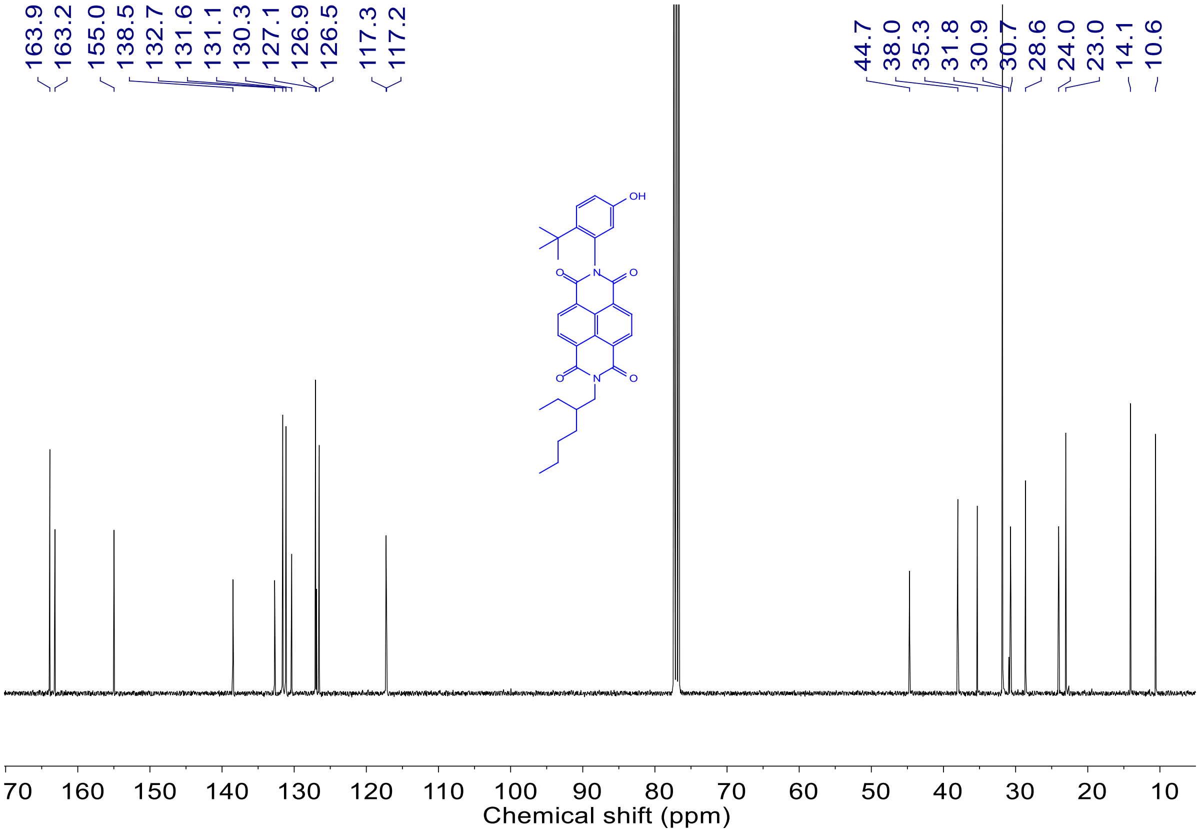

Fig. S9. ^{13}C NMR spectrum of **19** in $CDCl_3$.

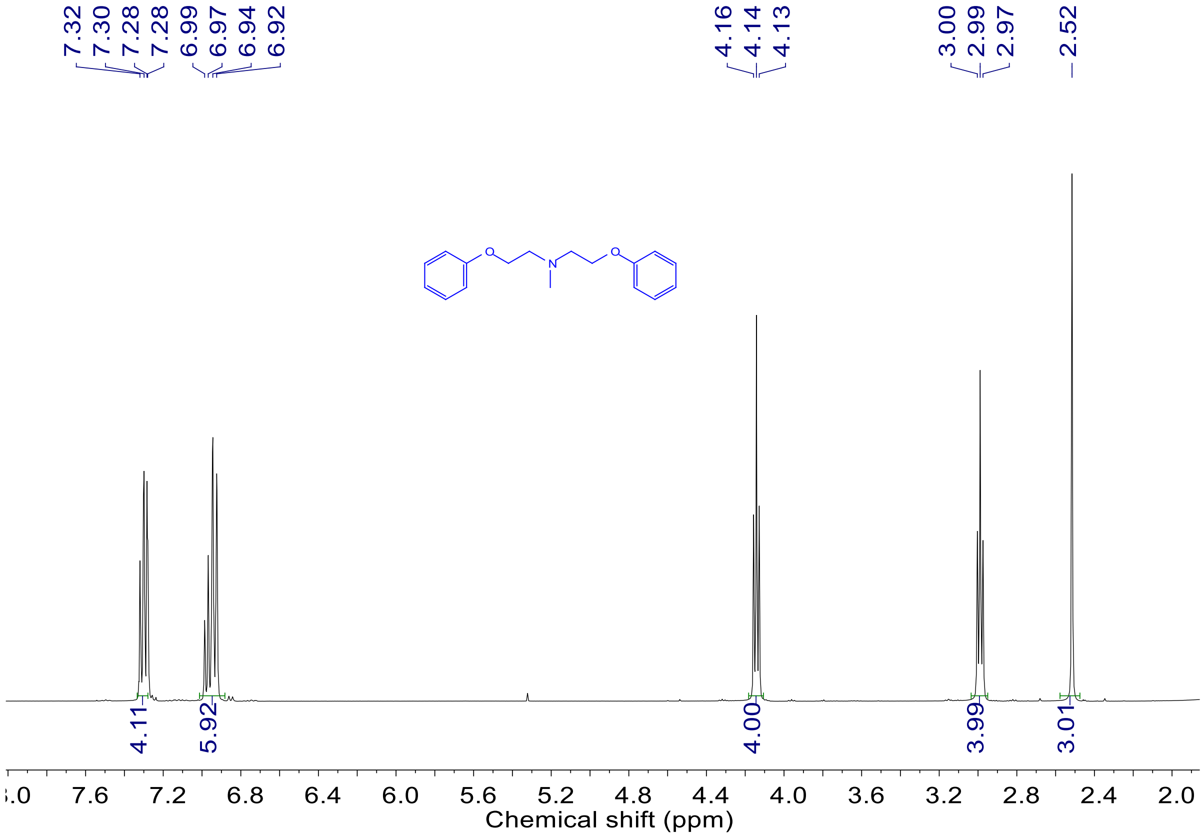

Fig. S10. ^{1}H NMR spectrum of **17** in $CDCl_3$.

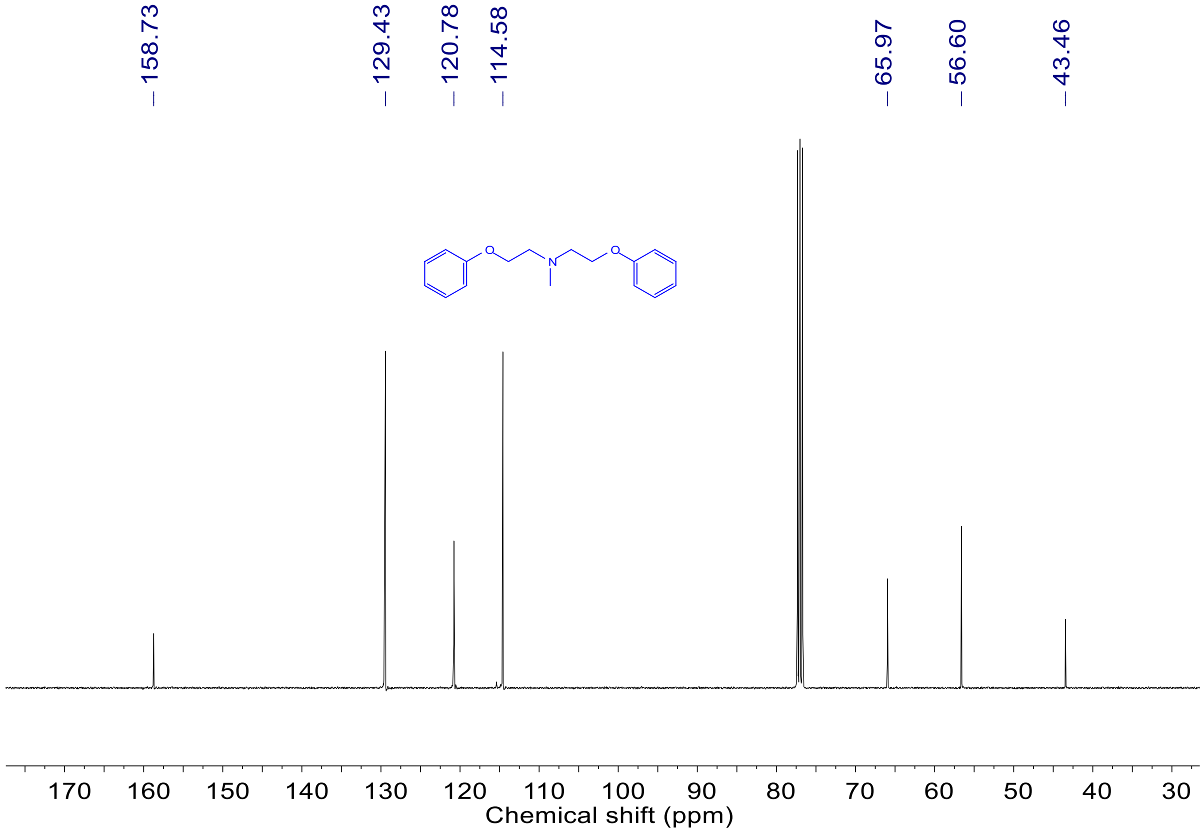

Fig. S11. ^{13}C NMR spectrum of **17** in $CDCl_3$.

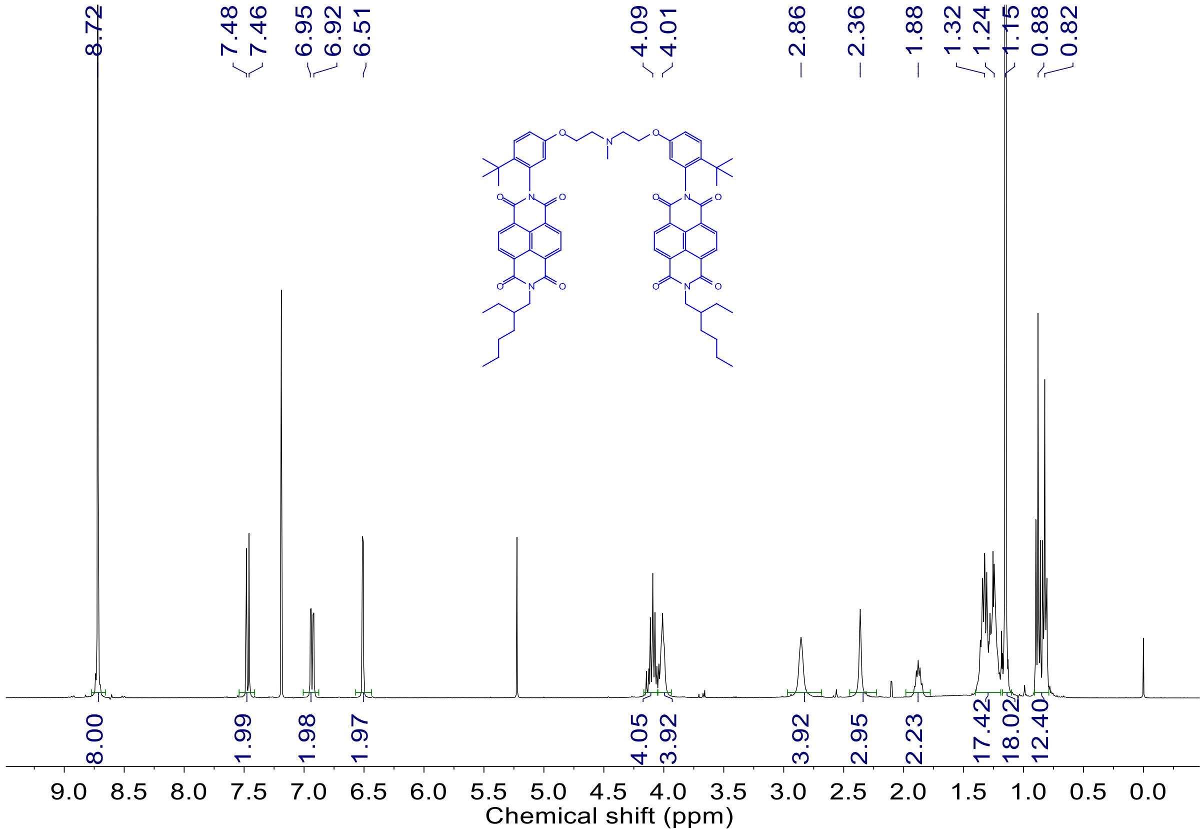

Fig. S12. ^{1}H NMR spectrum of **13** in $CDCl_3$.

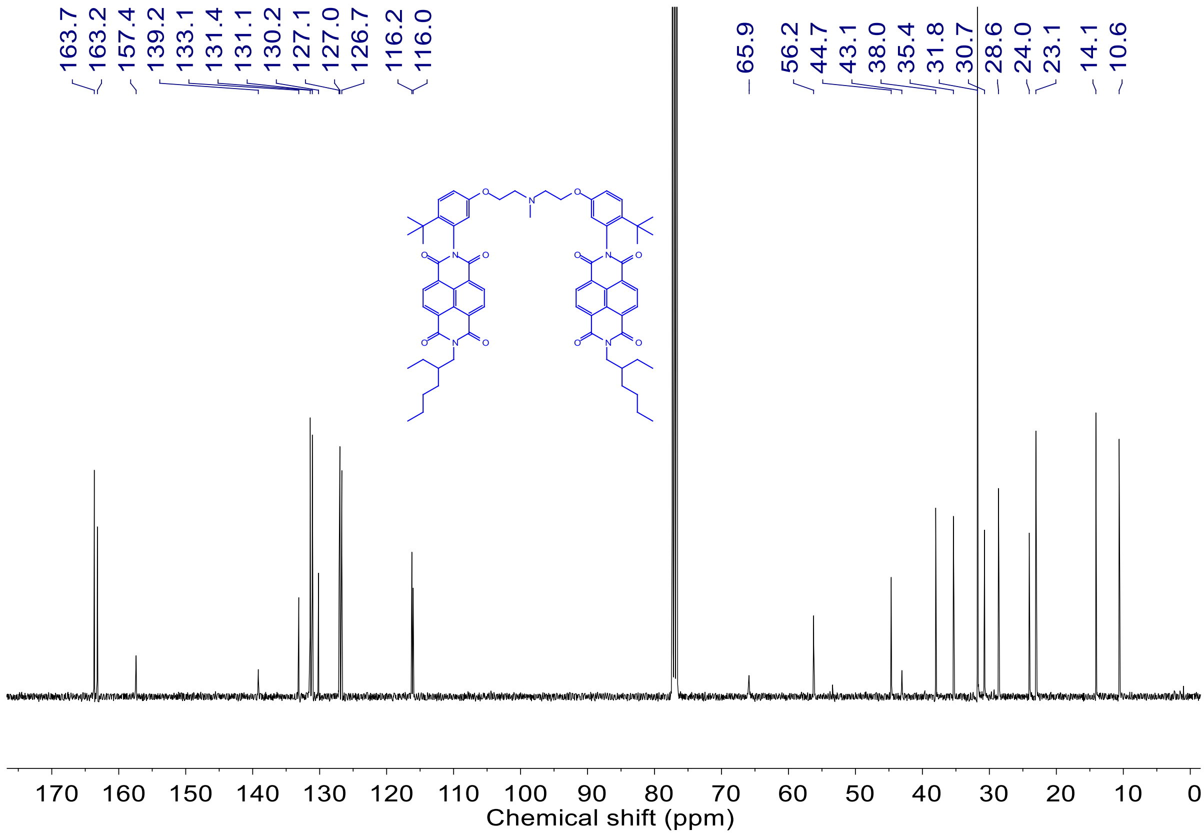

Fig. S13. ^{13}C NMR spectrum of **13** in $CDCl_3$.

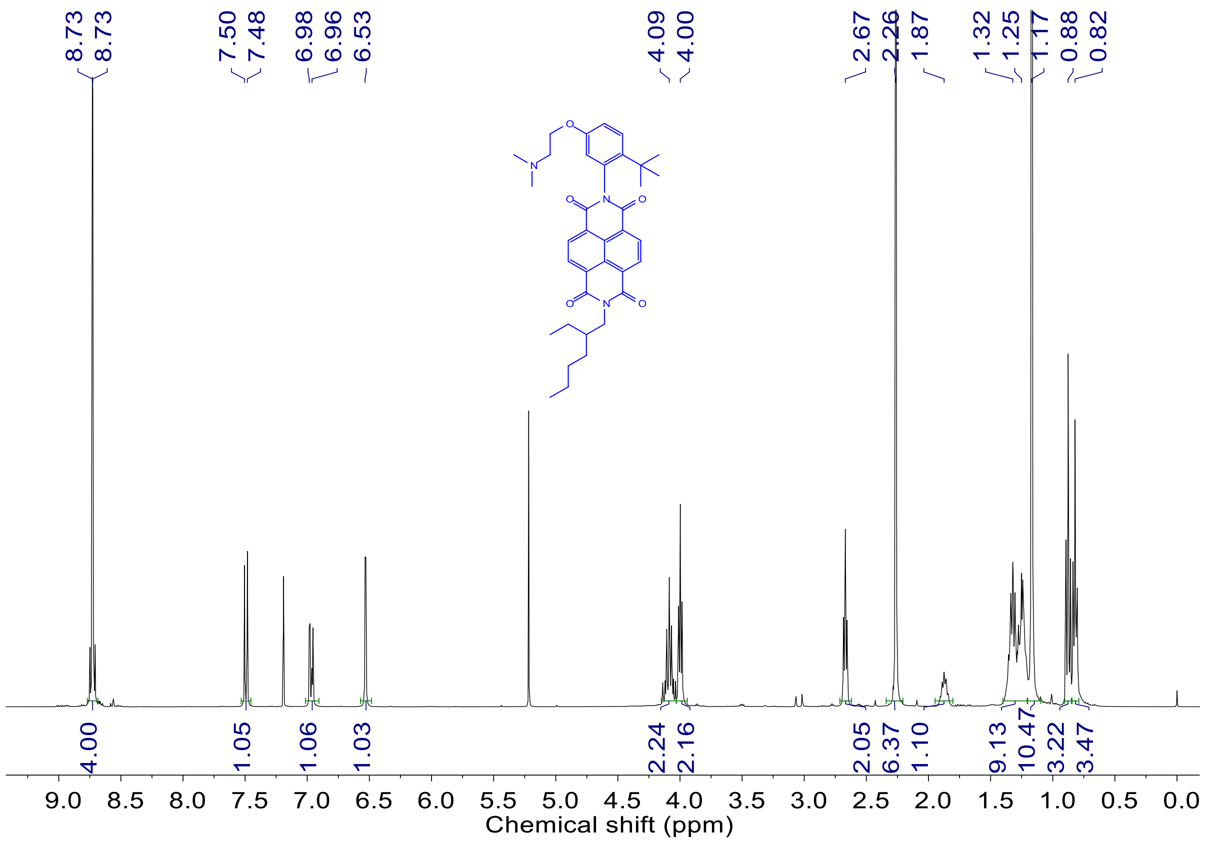

Fig. S14. ^{1}H NMR spectrum of **12** in $CDCl_3$.

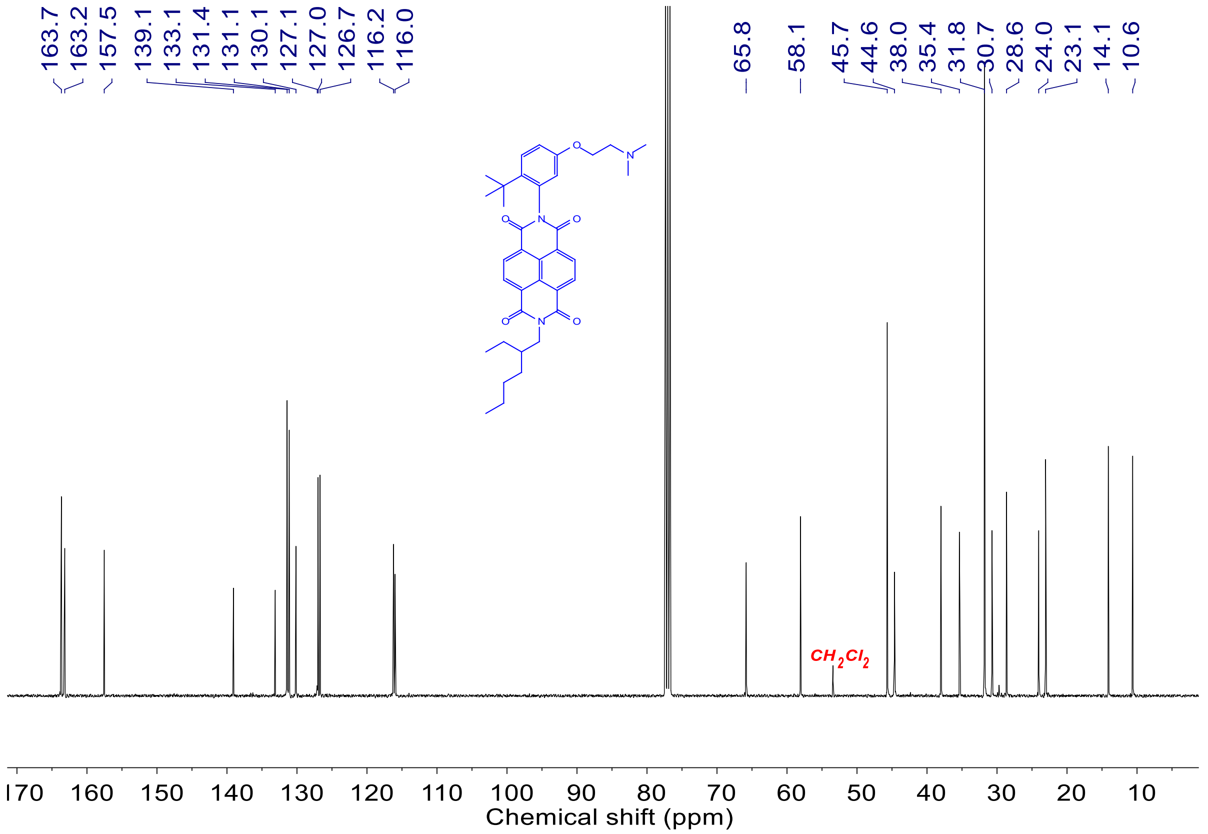

Fig. S15. ^{13}C NMR spectrum of **12** in $CDCl_3$.

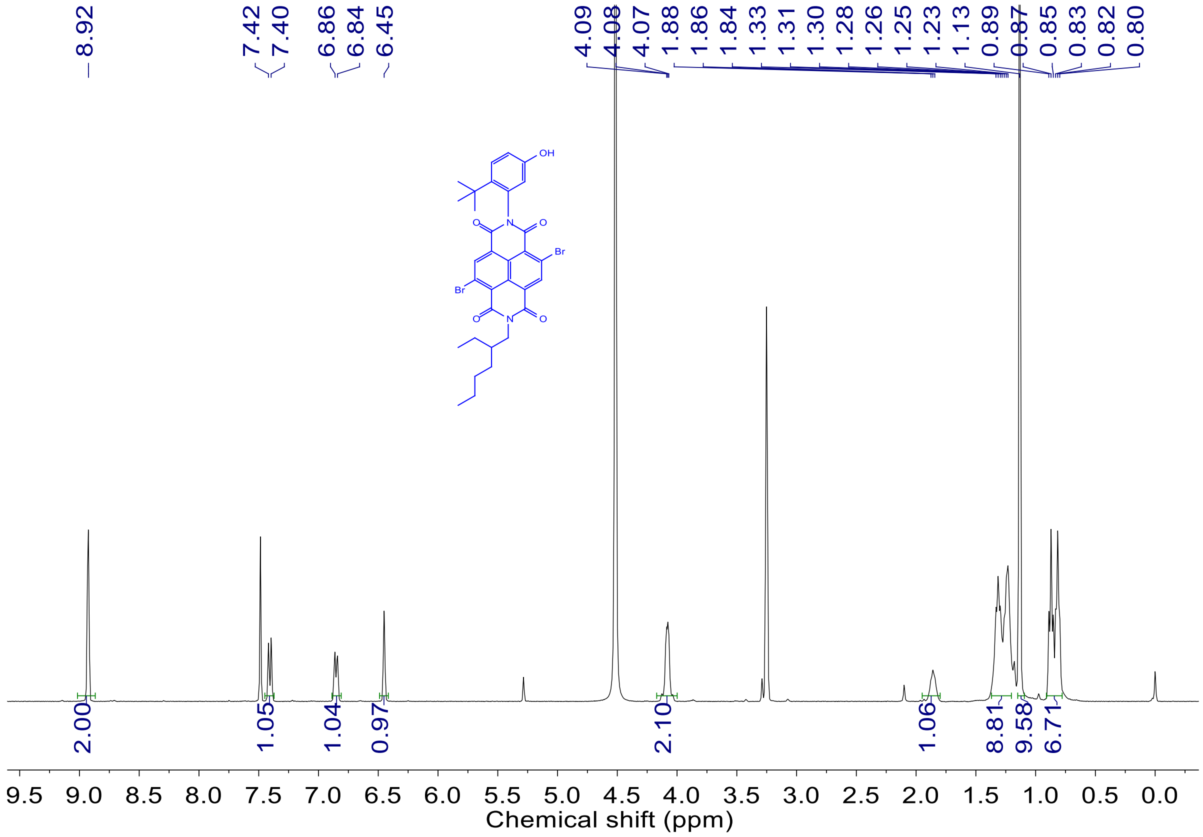

Fig. S16. ^{1}H NMR spectrum of **23** in $CDCl_3/CD_3OD$ 2:1.

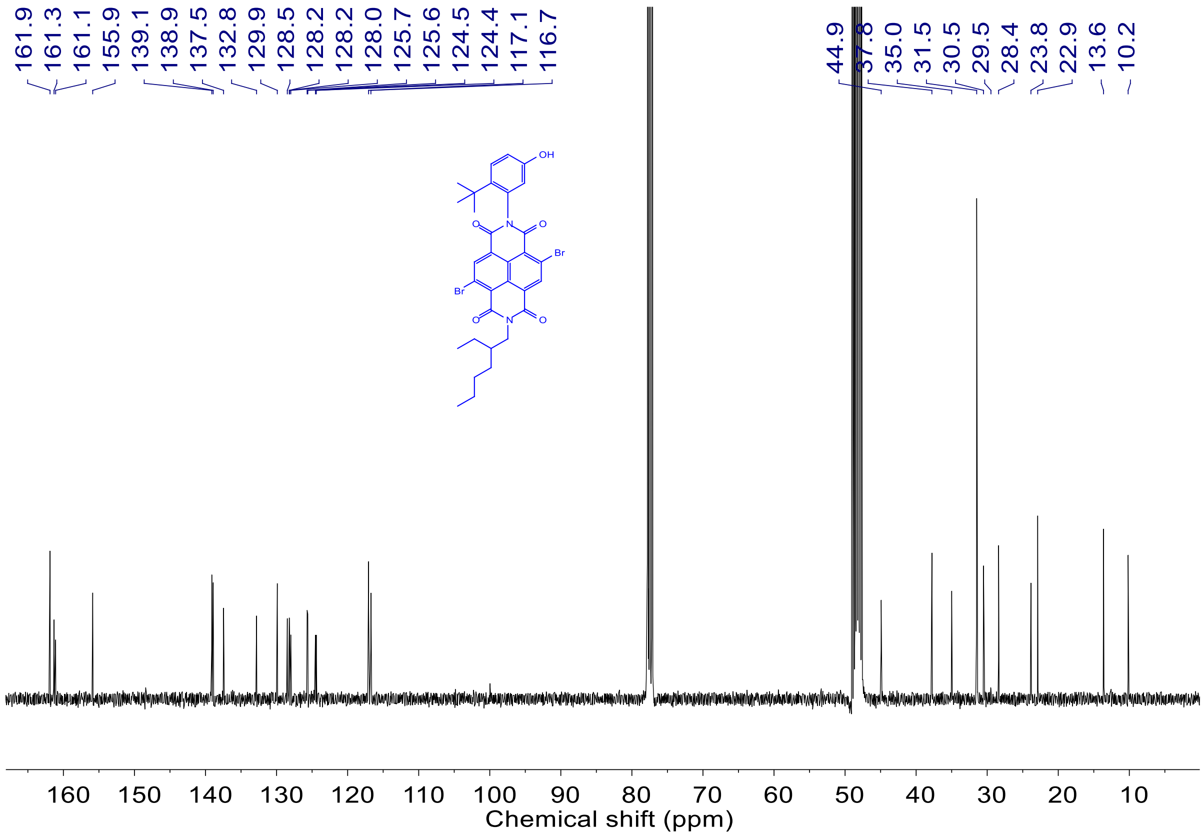

Fig. S17. ^{13}C NMR spectrum of **23** in $CDCl_3/CD_3OD$ 2:1.

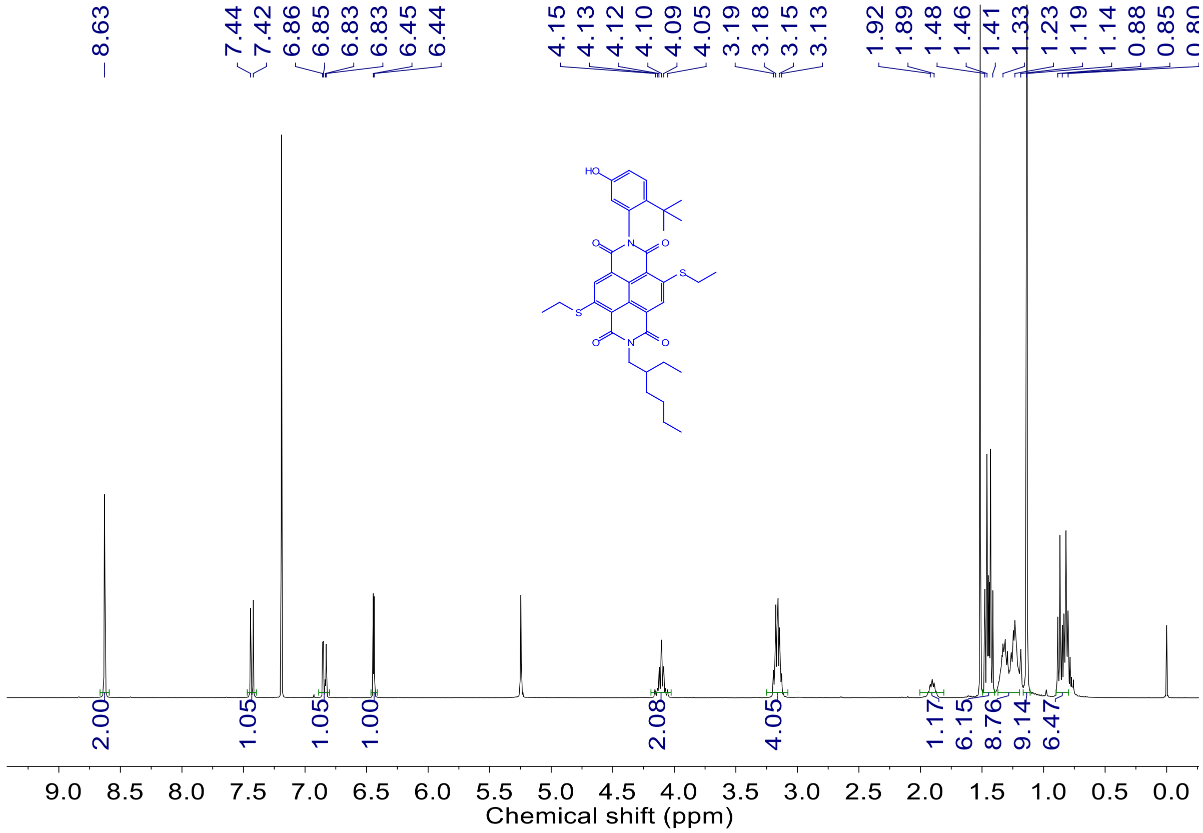

Fig. S18. ^{1}H NMR spectrum of **24** in $CDCl_3$.

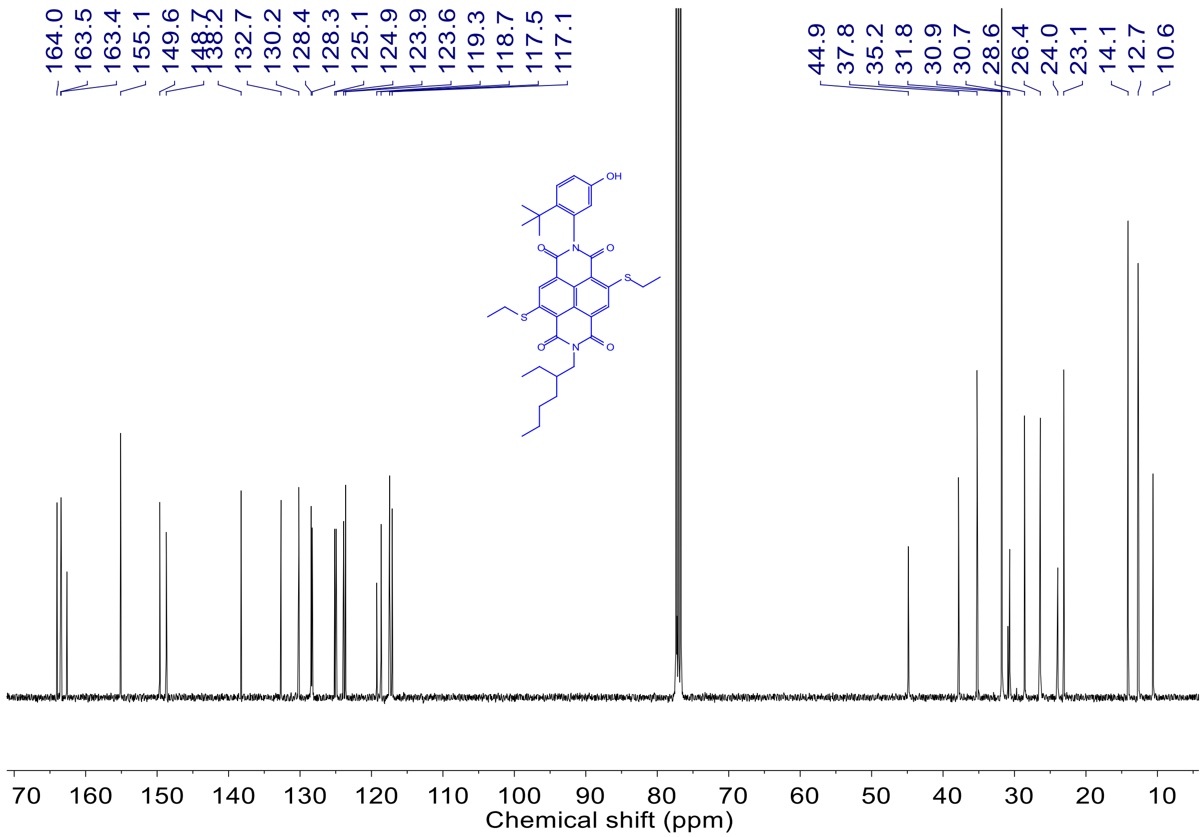

Fig. S19. ^{13}C NMR spectrum of **24** in $CDCl_3$.

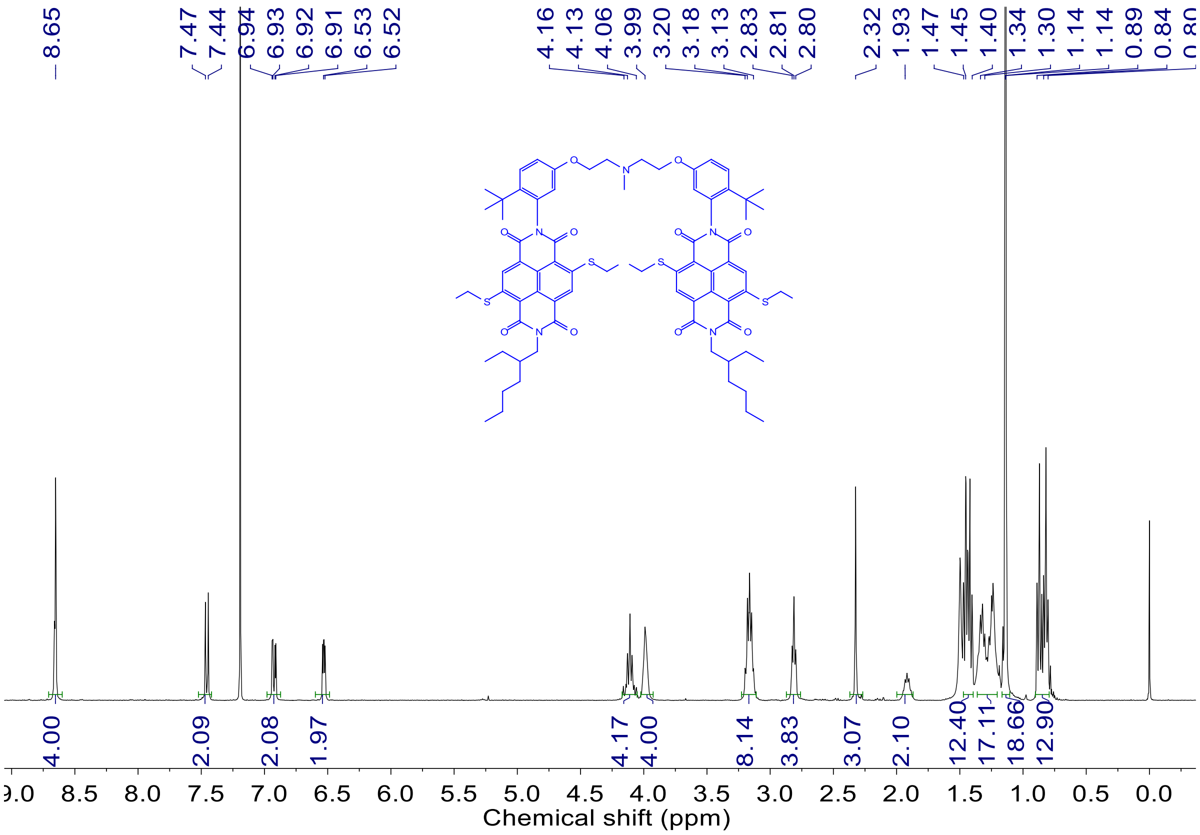

Fig. S20. ^{1}H NMR spectrum of **14** in $CDCl_3$ (mixture of stereoisomers).

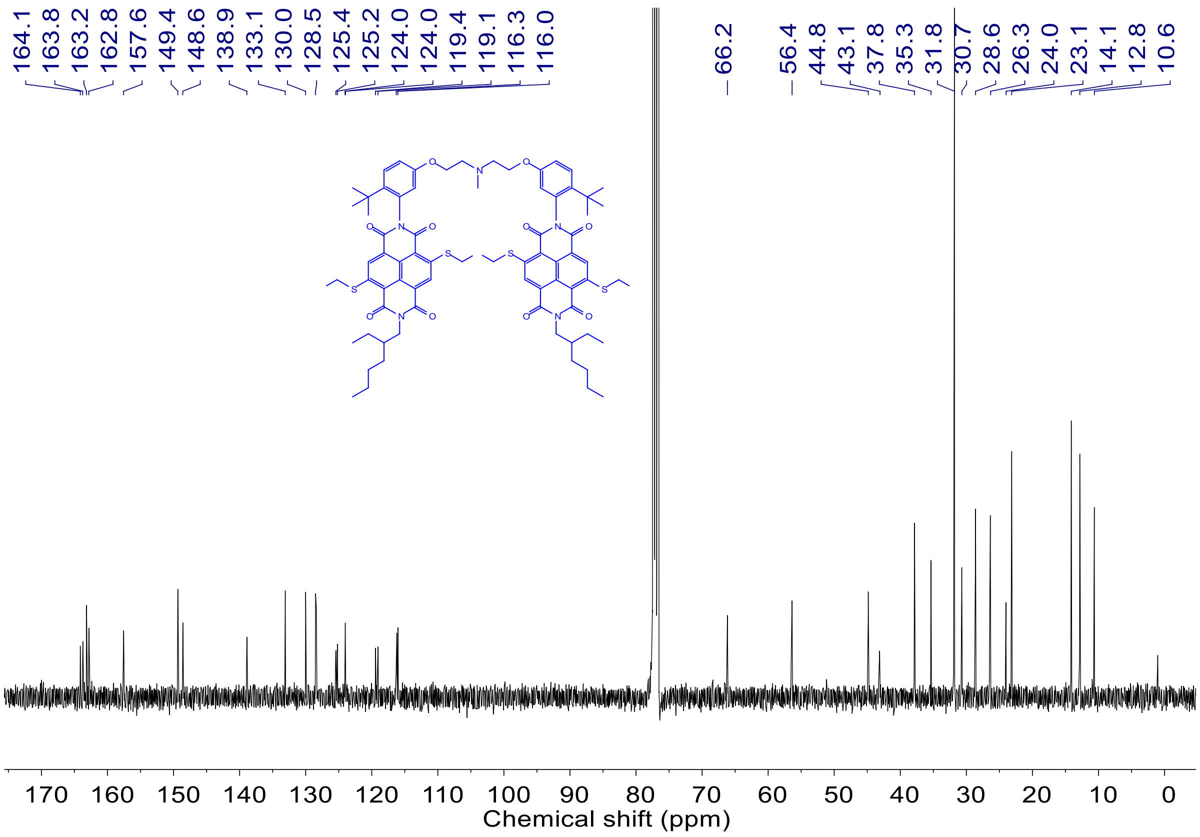

Fig. S21. ^{13}C NMR spectrum of **14** in $CDCl_3$ (mixture of stereoisomers).

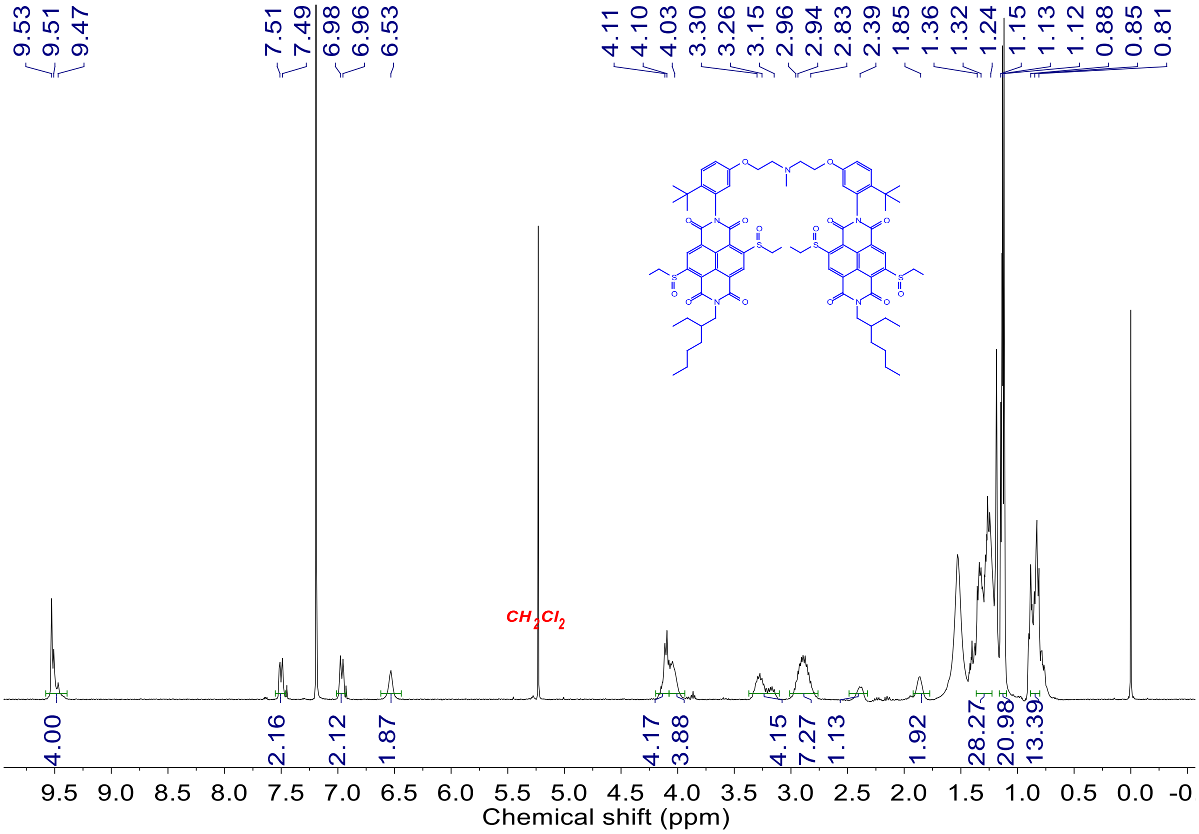

Fig. S22. ^{1}H NMR spectrum of **15** in $CDCl_3$ (mixture of stereoisomers, including sulfoxide chirality).

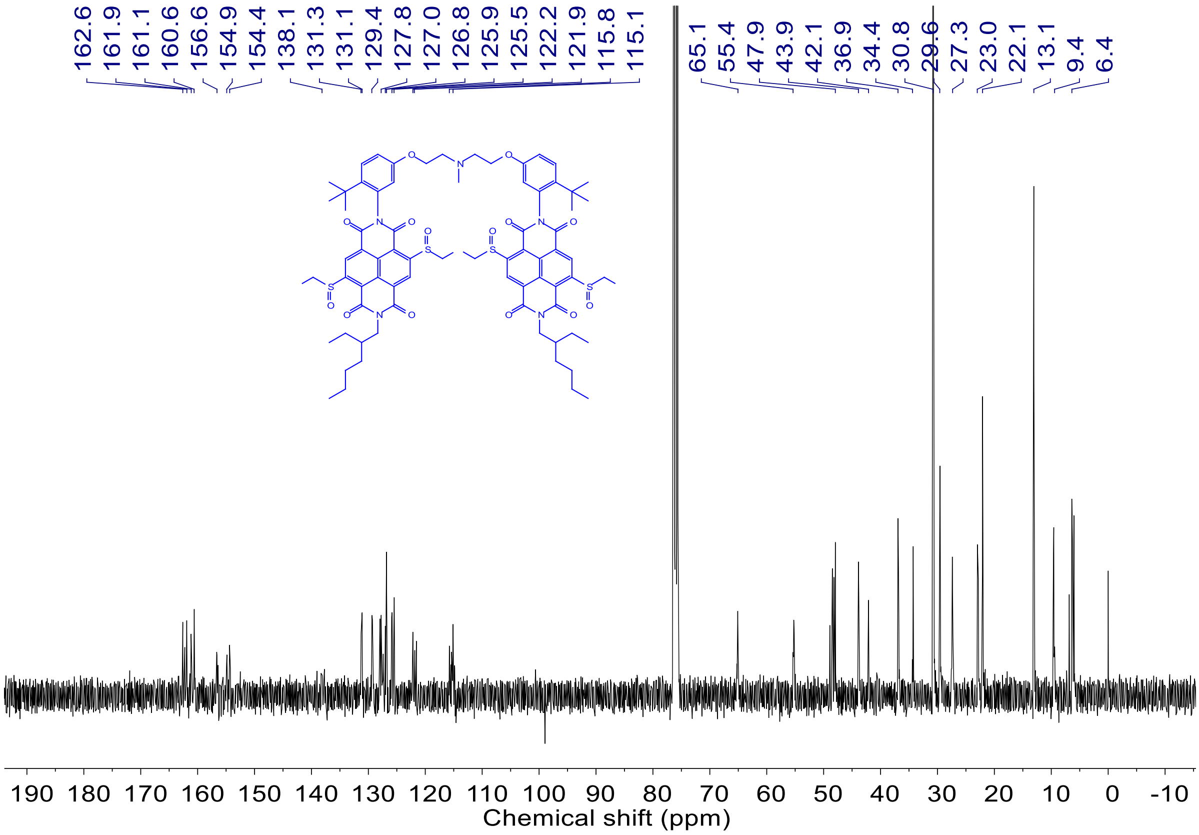

Fig. S23. ^{13}C NMR spectrum of **15** in $CDCl_3$ (mixture of stereoisomers, including sulfoxide chirality).

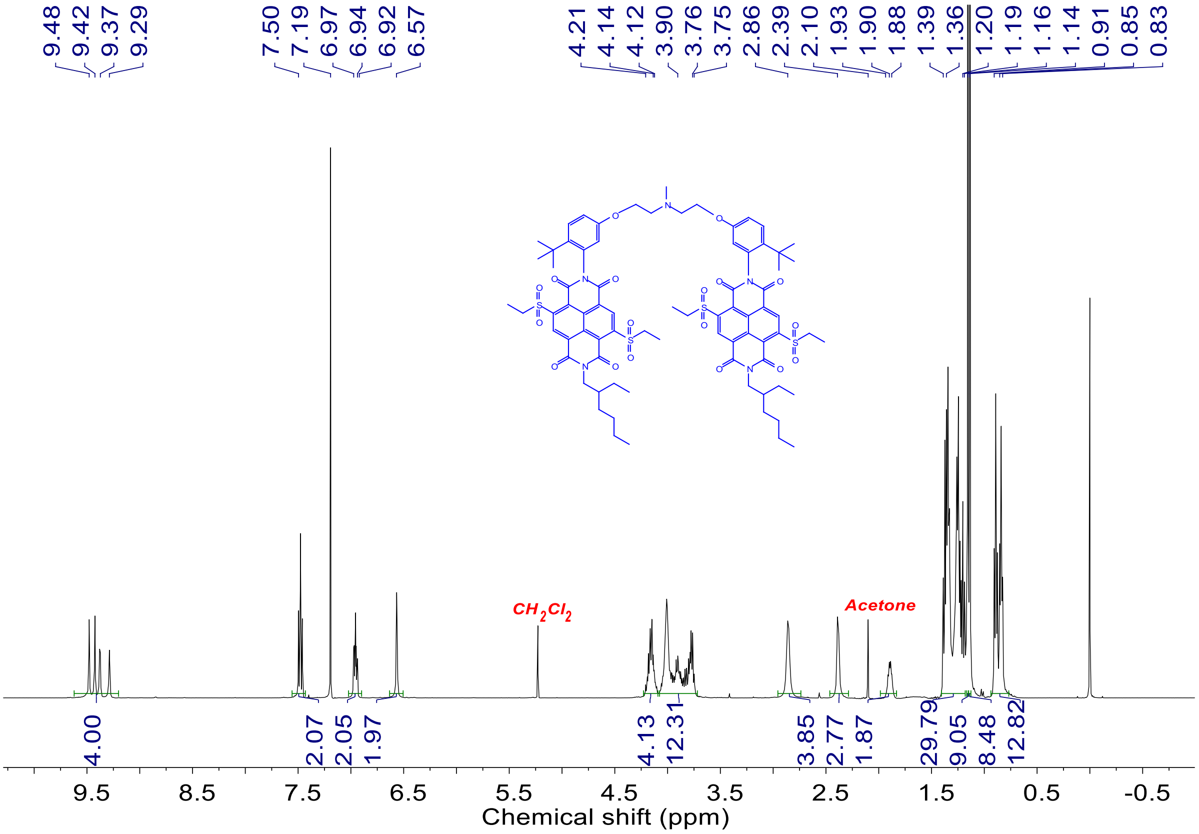

Fig. S24. ^{1}H NMR spectrum of **16** in $CDCl_3$ (mixture of stereoisomers).

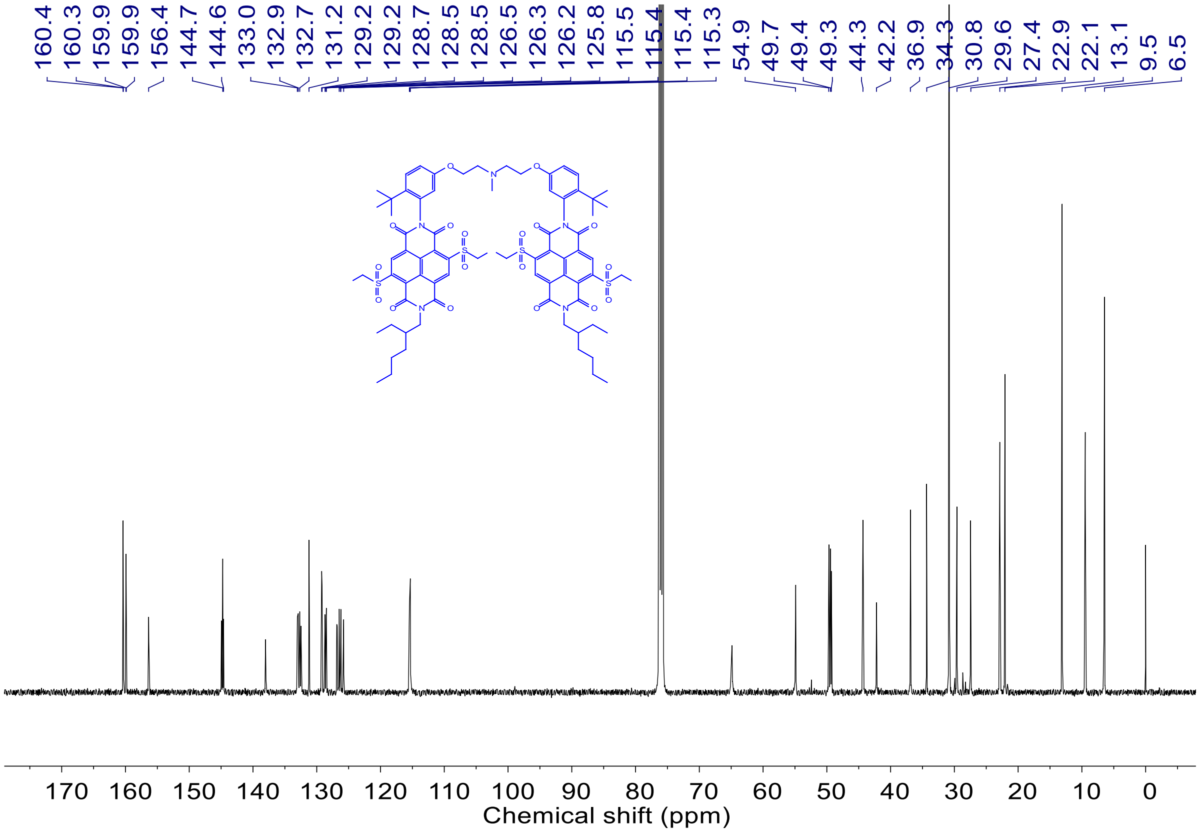

Fig. S25. ^{13}C NMR spectrum of **16** in $CDCl_3$ (mixture of stereoisomers).
